# Chidamide induces ROS-driven ZAKα-dependent ribotoxic stress in endometrial cancer and enhances radiosensitivity in vitro and in vivo

**DOI:** 10.1038/s41419-026-09125-x

**Published:** 2026-07-23

**Authors:** Haocheng Zhang, Zhiling Wang, Xinqin He, Yulong Mu, Xiaonan Fan, Xuan Wei, Hongluan Mao, Shuqi Chi, Zunling Li, Xuehui Pang, Wei Lu, Beihua Kong, Qing Zhang, Yingxin Pang

**Affiliations:** 1 Department of Obstetrics and Gynecology, Qilu Hospital of Shandong University, Jinan, China; 2 Shandong Engineering Laboratory for Urogynecology, Gynecologic Oncology Key Laboratory of Shandong Province, Qilu Hospital of Shandong University, Jinan, China; 3 Shandong Key Laboratory of Reproductive Health and Birth Defects Prevention and Control, Jinan, China; 4 Department of Obstetrics and Gynecology, The First Affiliated Hospital of Fujian Medical University, Fuzhou, China; 5 Department of Clinical Medicine, Medical School of Shandong University, Jinan, China; 6 Shandong Key Lab of Complex Medical Intelligence and Aging, Shandong Medicine and Health Key Lab of Respiratory Infection and Tumor Immunity, Department of Biochemistry and Molecular Biology, Shandong Tumour Immunotherapy Research Innovation Team, Binzhou Medical University, Yantai, China; 7 School of Pharmaceutical Sciences, Tiangong University, Tianjin, China

**Keywords:** Endometrial cancer, Cancer therapy, Apoptosis

## Abstract

Chidamide is a selective histone deacetylase inhibitor with antitumor activity in several malignancies, but its mechanism of action in endometrial cancer (EC) remains poorly understood. In this study, we investigated the cytotoxic effects and underlying mechanism of chidamide in EC using cell lines, patient-derived organoids (PDOs), cell-derived xenograft (CDX) models, and patient-derived xenograft (PDX) models. Chidamide markedly inhibited EC cell growth and induced reactive oxygen species (ROS) accumulation, DNA damage, G2/M-phase arrest, and apoptosis. Mechanistically, transcriptomic and functional analyses showed that chidamide disrupted translational homeostasis and activated a ROS-dependent, ZAKα-mediated ribotoxic stress response, leading to p38 and JNK activation. Scavenging ROS with N-acetylcysteine (NAC) attenuated ZAKα–MAPK signaling, and partially rescued chidamide-induced cell cycle arrest and apoptosis. Similarly, pharmacological ZAK inhibition or ZAKα silencing weakened stress signaling and diminished the cytotoxic effects of chidamide, supporting the central role of the ROS–ZAKα–MAPK axis. Chidamide also activated the GCN2–eIF2α–ATF4 integrated stress response, and ISRIB further enhanced stress signaling and apoptosis, suggesting an adaptive buffering role of this pathway. In addition to its activity in vitro, chidamide showed broad antitumor efficacy in PDO, CDX, and PDX models. Chidamide also enhanced tumor radiosensitivity in vivo, supporting its potential utility in combination with radiotherapy. Together, these findings identify ROS-driven, ZAKα-dependent ribotoxic stress signaling as a key mechanism underlying chidamide-induced cytotoxicity in EC and provide a mechanistic rationale for the further development of chidamide-based strategies in this disease.

**Figure:**
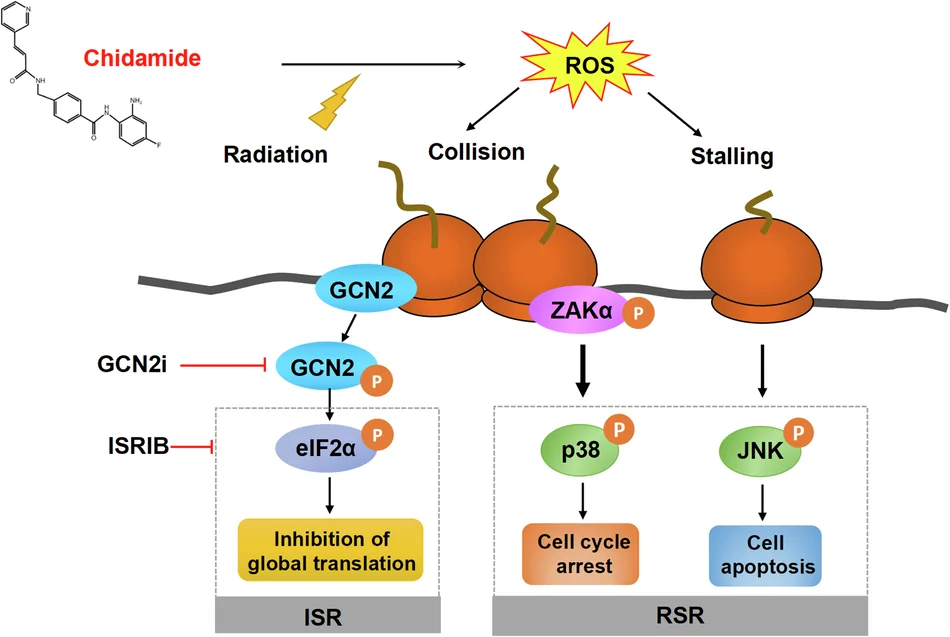

## Background

Endometrial cancer (EC) is the most common malignant gynecological tumor in developed countries; its incidence has continued to increase in recent decades, and there is a similar upward trend worldwide [1, 2]. Although surgery is still the cornerstone of radical treatment, patients with high-risk EC still face a high recurrence rate and poor long-term prognosis even after undergoing radiotherapy and systemic treatment [3]. In particular, the survival rate of late-stage patients is still very low, which highlights the urgent need to develop more effective treatment strategies for patients with high-risk EC.

Given the urgent need for novel therapeutic strategies in EC, epigenetic therapies have emerged as a promising area of investigation. Histone deacetylase inhibitors (HDACis) are a class of epigenetic drugs with a wide range of antitumour activities. Chidamide is a selective HDACi that targets class I HDACs (HDAC1, HDAC2, and HDAC3) and HDAC10. Compared with first-generation pan-HDACis, such as vorinostat and trichostatin A, chidamide has greater subtype selectivity and better therapeutic effects. In addition to its established clinical activity in malignant tumors of the blood system [4], chidamide has been shown to have good antitumor effects on a variety of solid tumors, including breast cancer [5], colorectal cancer [6], and non-small cell lung cancer [7]. Previous studies have shown that chidamide can induce cell cycle arrest, apoptosis, autophagy, and antiangiogenic activity [8, 9] and may enhance antitumor immunity when combined with the anti-PD-1 antibody toripalimab [10]. However, the therapeutic potential mechanism of chidamide in EC is unknown.

Recent evidence has shown that in addition to its classic epigenetic effects, chidamide may affect ribosome biogenesis and stress signaling. In acute myeloid leukemia, chidamide disrupts ribosome biogenesis by interfering with c-MYC activity [11]. Ribosome stalling and collision are considered key stress perception events triggered by various types of cell damage, including UV [12] and genotoxic damage [13], and their mechanisms of action are being increasingly emphasized. Mild ribosomal stress can activate the integrated stress response (ISR), leading to the global suppression of protein synthesis [14–16], while ribotoxic stress is perceived by MAP3K ZAKα, which in turn activates the ribotoxic stress response (RSR) and the downstream p38 and JNK signaling pathways, ultimately leading to cell cycle arrest [17, 18] and apoptosis [19].

However, whether the stress response pathway is involved in the cytotoxicity of chidamide in EC is unclear. In this study, we first investigated the molecular basis of chidamide-induced cytotoxicity in EC cell lines and further validated the key mechanistic findings in PDO model. In parallel, CDX and PDX models were used to assess the in vivo antitumor activity of chidamide and its ability to enhance tumor radiosensitivity. We found that chidamide inhibits the growth of EC cells by inducing a ROS-dependent RSR, thereby activating the ZAKα–MAPK signaling pathway and leading to cell cycle arrest and apoptosis. These findings reveal a previously unrecognized mechanism through which chidamide plays a role in EC and provide a theoretical basis for its therapeutic development, especially for the treatment of high-risk diseases.

## Material and methods

### Reagents

The chemicals and inhibitors used in this study were as follows: chidamide (Selleckchem, #S8567), puromycin (InvivoGen, #ant-pr-1), N-Acetyl-L-cysteine (NAC, MedChemExpress, #HY-B0215), harringtonine (HTN, Selleckchem, #S9063), ZAK inhibitor (gifted from Xiaoyun Lu, Jinan University, Guangzhou, China), ISRIB (Sigma-Aldrich, #SML0843), GCN2 inhibitor A-92 (Selleckchem, #E2987) and PERK inhibitor GSK2606414 (SelleckChem, #S7307), collagenase IV (Sangon Biotech, #A004186), ROCK inhibitor Y-27632 (Selleck, #S1049), Matrigel (Corning, #356231).

### Cell lines and cell culture

AN3CA, HEC-1A, and RL95-2 cells were obtained from the Chinese Academy of Sciences, and Ishikawa cells were sourced from the ECACC (European Collection of Authenticated Cell Cultures). AN3CA cells were maintained in MEM (Gibco, #11095080) supplemented with 1 mM sodium pyruvate (Gibco, #11360070), 1 × NEAA (Gibco, #11140050), and 10% FBS (Gibco, #A5256701). HEC-1A cells were propagated in McCoy’s 5A (Gibco, #16600082) containing 1× sodium pyruvate and 10% FBS. Ishikawa cells were cultured in DMEM (Gibco, #11965092) with 10% FBS, whereas RL95-2 cells grew in DMEM/F12 (Gibco, #11320033) with 0.005 mg/mL insulin and 10% FBS. All cultures were maintained in an incubator with 5% CO₂ at 37 °C. All cell lines were authenticated by short tandem repeat (STR) profiling before experimental use. Mycoplasma contamination was routinely tested and excluded.

### TCGA database analysis

RNA-seq expression data and corresponding clinical information for EC were obtained from The Cancer Genome Atlas (TCGA) database. Differential expression and survival analyses were performed using the Xiantao Academic online tool (https://www.xiantaozi.com/). For survival analysis, patients were divided into high- and low-expression groups according to the optimal cutoff value provided by the platform. Kaplan–Meier survival curves were generated, and differences between groups were compared using the log-rank test. Overall survival (OS) and progression-free survival (PFS) were used as the survival endpoints.

### Tissue specimens

Primary EC tissues were obtained from the Department of Obstetrics and Gynecology, Qilu Hospital, Shandong University, after written informed consent was provided by the patients. All aspects of this study were approved by the Ethics Committee on Scientific Research of Shandong University, Qilu Hospital, Jinan (Approval No. KYLL-202409-048).

### Western blot

Whole-cell lysates were prepared by RIPA buffer (Thermo Scientific, #89900) with protease inhibitors (APExBIO, #K1007) and phosphatase inhibitors (APExBIO, #K1015), then lysates were centrifuged at 4 °C using a filtration column (NCMBiotech, #WB3050). Concentrations of protein were detected by a BCA kit (Thermo Scientific, #23225). Proteins were mixed with loading buffer (Beyotime, #P0015) and denatured by boiling for 5 min. The samples were separated by SDS-PAGE and transferred onto PVDF membranes (Millipore, #GVHP00010). Membranes were blocked in TBST containing 5% skim milk at room temperature (RT) for 1 h to prevent non-specific binding, followed by incubation with primary antibodies at 4 °C. The next day, membranes were washed three times with TBST for 10 min each. The membrane was incubated with HRP-conjugated secondary antibodies for 1 h at RT and washed again for 3 times. Lastly, membranes were visualized by Image Quant LAS4010 (GE Healthcare) with ECL Ultra Western HRP substrate (Millipore, #WBULS0100). Detailed information about the antibodies is in Supplementary Table 1.

### Cell viability analysis

EC cells were seeded into 96-well plates at a density of 3000–5000 cells per well and exposed to increasing concentrations of chidamide or increasing doses of radiation. 10% CCK-8 solution (MeilunBio, #MA0225) in fresh medium was added to each well, with blank wells receiving CCK-8-containing medium alone as background controls. After incubation at 37 °C for 2 h, absorbance was measured at 450 nm using a microplate spectrophotometer (Thermo Scientific, USA).

### Cell apoptosis assays

Apoptosis was detected by flow cytometry using Annexin V/7-AAD apoptosis detection kit (GOONIE, #100-105). Cells were detached with Accutase (Sigma-Aldrich, #A6964), washed with PBS, and resuspended in 500 μL 1 × Binding Buffer. Each sample was incubated with 5 μL Annexin V and 5 μL 7-AAD for 10 min at RT in the dark. Stained cells were acquired on a Beckman CytoFLEX flow cytometer (Beckman Coulter), and data were analyzed with FlowJo v10.4.

### Cell cycle assays

For cell cycle, cells were processed using the Cell Cycle Kit (MultiSciences, #CCS012). After permeabilization, cells were stained with PI and analyzed by flow cytometry to determine phase distribution. Data were analyzed with FlowJo v10.4.

### ROS detection assays

Intracellular ROS levels were measured using DCFH-DA (Beyotime, #CS0033). The probe was diluted 1:1000 in PBS to a final concentration of 10 μM. After removal of the culture medium, cells were incubated with the diluted DCFH-DA solution (at least 1 mL per well in a 6-well plate) at 37 °C for 20 min. Cells were then washed three times with extracellular solution. Fluorescence was analyzed by flow cytometry.

### Comet assay

DNA damage was detected using the comet assay (Beyotime, #C2041). After the indicated treatments, cells were collected, washed with PBS, and resuspended at approximately 1 × 10^6^ cells/mL. Cells were mixed with 0.7% low-melting-point agarose and applied onto slides precoated with 1% normal-melting-point agarose. After solidification at 4 °C, slides were lysed in cold lysis solution (lysis buffer:DMSO = 9:1) at 4 °C overnight. Following lysis, slides were incubated in prechilled alkaline electrophoresis buffer for 20 min and subjected to electrophoresis at 25 V for 20 min in the dark. Slides were then neutralized, stained with PI, and imaged under a fluorescence microscope. Comet parameters were quantified using CASP software.

### Immunofluorescence

The experiment used a detection kit (Beyotime, #C2035S). Cells cultured on glass slides were fixed in 4% paraformaldehyde for 15 min at RT and permeabilized with 0.2% Triton X-100 for 5 min. After blocking with normal goat serum for 30 min at RT, the cells were incubated overnight at 4 °C with primary antibodies against γ-H2AX, followed by incubation with the appropriate secondary antibodies for 1 h at 37 °C. Nuclei were counterstained with DAPI for 5 min at RT. Slides were mounted with antifade medium and sealed with coverslips. Fluorescence images were captured using a confocal microscope (Sunny).

### RNA-seq and data analysis

For RNA sequencing, HEC-1A and Ishikawa cells were treated with either DMSO or chidamide (5 μM) for 24 h. Total RNA was collected using TRIzol, with three independent biological replicates prepared for each condition. Differentially expressed genes (DEGs) between the two groups were identified using the limma-voom R package. Genes with a *P* value < 0.05 and an absolute log_2_ fold change (|log_2_FC|) > 1 were considered significantly differentially expressed. Biological process enrichment analysis was executed utilizing the clusterProfiler R package. Gene Ontology (GO) enrichment analysis and Kyoto Encyclopedia of Genes and Genomes (KEGG) enrichment analysis were performed to identify enriched biological processes and pathways.

### SUrface SEnsing of Translation (SUnSET) assays

Cells were treated with the indicated reagents, followed by the addition of puromycin (10 μg/mL; InvivoGen, #ant-pr-1) to the culture medium for the specified duration. To ensure consistency, all samples were exposed to the same puromycin concentration for an identical incubation period. After treatment, cells were washed with cold PBS and immediately lysed. The incorporation of puromycin into nascent polypeptide chains was detected by Western blot using anti-puromycin antibodies.

### Argyrophilic nucleolar organizer region (AgNOR) staining

AgNOR staining was used to assess ribosome biogenesis using a commercial kit (Saint-Bio, #R23062). The staining solution was freshly prepared by mixing solution A and solution B at a 1:1 ratio. After fixation, cells were incubated with the staining solution and reacted for 30 min at RT. The cells were washed twice with PBS to remove excess dye and imaged by microscope (Olympus). Quantification of AgNOR staining was performed using ImageJ. All experiments were conducted in three independent biological replicates.

### Cell transfection

siRNAs and negative control (siNC) targeting ZAKα were bought from GenePharma (Shanghai, China). Transfections of siRNA/siNC were carried out by Lipofectamine 2000 (Invitrogen, #11668019). For siRNA transfection, cells were seeded into 6-well plates at approximately 2.5 × 10^5^ cells per well. Transfection was performed after 12 h, when cells reached 30–50% confluence. Cells were incubated for 48 h after transfection before subsequent experiments were performed. Sequences were: si1-ZAKα: 5’- GAAGUGGAUUGUAGGAAUA (dTdT); si2-ZAKα: 5′- CCCAGAGCAAUCCUAUUCU (dTdT); si3-ZAKα: 5′- CAGCAUUCCACUCCUUCAA (dTdT).

### Organoid culture

Fresh EC tissues were minced with sterile scissors, washed repeatedly in Ca²⁺/Mg²⁺-free PBS, and digested in collagenase IV (2 mg/mL) supplemented with the ROCK inhibitor Y-27632 (10 µM), then filtered through a 100 µm cell strainer. The filtrate was centrifuged, and the pellet was optionally treated with red blood cell lysis buffer. After centrifugation and one wash with DPBS, cells were resuspended in a mixture of 60% Matrigel and 40% advanced DMEM/F12. Aliquots of 30 µL were plated as droplets in prewarmed 24-well plates and overlaid with 500 µL of organoid culture medium (bioGenous, #K2166-EC), which was refreshed every 5 days. For passaging (every 10–20 days), Matrigel was dissolved by incubating organoids in Cell Recovery Solution (Corning, #354253) at 4 °C for 30 min. Organoids were gently collected using BSA-coated pipette tips and tubes, then dissociated with enzymatic dissociation solution (bioGenous, #E238001) at 37 °C for 7 min. The reaction was quenched by diluting the mixture fivefold with culture medium. The resulting single cell or small cluster suspension was used for replanting, cryopreservation, or downstream experiments.

### Immunohistochemistry (IHC) and scoring

Paraffin sections were deparaffinized in xylene, rehydrated through a graded ethanol series, and subjected to antigen retrieval with either citrate buffer (pH 6.0) or Tris-EDTA buffer (pH 9.0). Endogenous peroxidase activity was quenched with 3% H₂O₂, and non-specific binding was blocked using 5% goat serum. Sections were then incubated overnight at 4 °C with primary antibodies against Ki-67 (1:1500), p-p38 (1:600), or p-JNK (1:600), followed by an HRP-conjugated secondary antibody. Immunoreactivity was visualized using a DAB kit (Proteintech, #PR30018). Staining was assessed semi-quantitatively by combining the percentage of positive tumor cells and staining intensity. The percentage score was defined as follows: 0 (<5%), 1 (5–25%), 2 (25–50%), 3 (50–75%), 4 (≥75%). Intensity was scored as follows: 0 (negative), 1 (weak, light yellow), 2 (moderate, yellow), or 3 (strong, brown). A final immunoreactivity score (range: 0–12) was obtained by multiplying the two scores.

### Synergy evaluation of chemo-radiotherapy in vitro

Cells were seeded in 96-well plates at an optimized density and allowed to adhere overnight. They were then treated with chidamide at specified concentrations and exposed to defined doses of X-ray irradiation. Cell viability was assessed using the CCK-8 assay following the manufacturer’s protocol. Drug–radiation interactions were analyzed with SynergyFinder v3.0, and combination effects were quantified using the Zero Interaction Potency (ZIP) model. A ZIP score <−10 indicated antagonism, −10 to 10 indicated additive effects, and >10 indicated synergism.

### Xenograft studies

Nude mice (BALB/c; female; 4–6 weeks old) were purchased from GemPharmatech Company (Jiangsu, China). For CDX model, HEC-1A cells (at 1.0 × 10^6^ cells per mouse) were injected subcutaneously into the right axillary region of nude mice. Treatment was initiated when the tumor volume reached approximately 75 mm³; each group included six mice. For PDX, NCG (NOD/ShiLtJGpt-Prkdcem26Cd52Il2rgem26Cd22/Gpt; female; aged 4–6 weeks) were also purchased from GemPharmatech Company. PDX models were established following previously described protocols. Briefly, freshly resected tumor specimens were minced into fragments approximately 1–2 mm³ in size and suspended in PBS containing 1 × penicillin-streptomycin-Amphotericin B (Thermo Scientific, #15240062). The tissue fragments were then taken subcutaneous implantation into the axillary region of NCG mice within 3 h of surgical collection. Successfully engrafted tumors were designated as passage 1 (P1). When P1 tumors reached a volume of approximately 750 mm³, mice were performed euthanasia, and tumor fragments were either implanted into mice to generate passage 2 (P2) or cryopreserved in 90% FBS and 10% DMSO for future use. P3 tumors were used for the subsequent experiments. When the tumor volumes reached about 75 mm^3^, mice were randomly assigned to four groups (five mice per group). The corresponding treatments were given: Vehicle, chidamide (25 mg/kg), X-ray (2 Gy), and combined usage of chidamide and X-ray. Medication and radiation treatments were administered on alternate days, and tumor volumes were monitored throughout the experiment. At the end, mice were euthanized, and tumors were taken out, photographed, weighed, and their dimensions recorded. All animal handling and experimental procedures followed the Guidelines for Animal Experiments of Shandong University and were reviewed and approved by the Committee on the Ethics of Animal Experiments of Shandong University, Jinan (Approval No. 24049).

### Statistical analysis

Statistical analyses were performed using GraphPad Prism 10.2.0. Differences between two groups were analyzed using an unpaired two-sided Student’s t-test. Comparisons among three or more groups were analyzed using one-way ANOVA followed by Tukey’s multiple comparisons test. Data are presented as mean ± SD from three independent experiments unless otherwise stated. Statistical significance was defined as *p* < 0.05 (ns not significant; *p < 0.05; **p < 0.01; ***p < 0.001; ****p < 0.0001).

## Results

### Chidamide induces cytotoxic stress, G2/M arrest, and apoptosis in endometrial cancer cells

To provide supporting evidence for HDAC-targeted therapy in EC, we assessed class I HDAC expression and clinical relevance. TCGA analysis and immunoblotting of clinical specimens showed increased class I HDAC expression in EC, while survival analysis indicated that elevated HDAC2 and HDAC3 expression was associated with poorer outcomes (Fig. S1A–F). These findings provided a rationale for evaluating chidamide, a selective class I HDACi, in EC models.

**Fig. 1:**
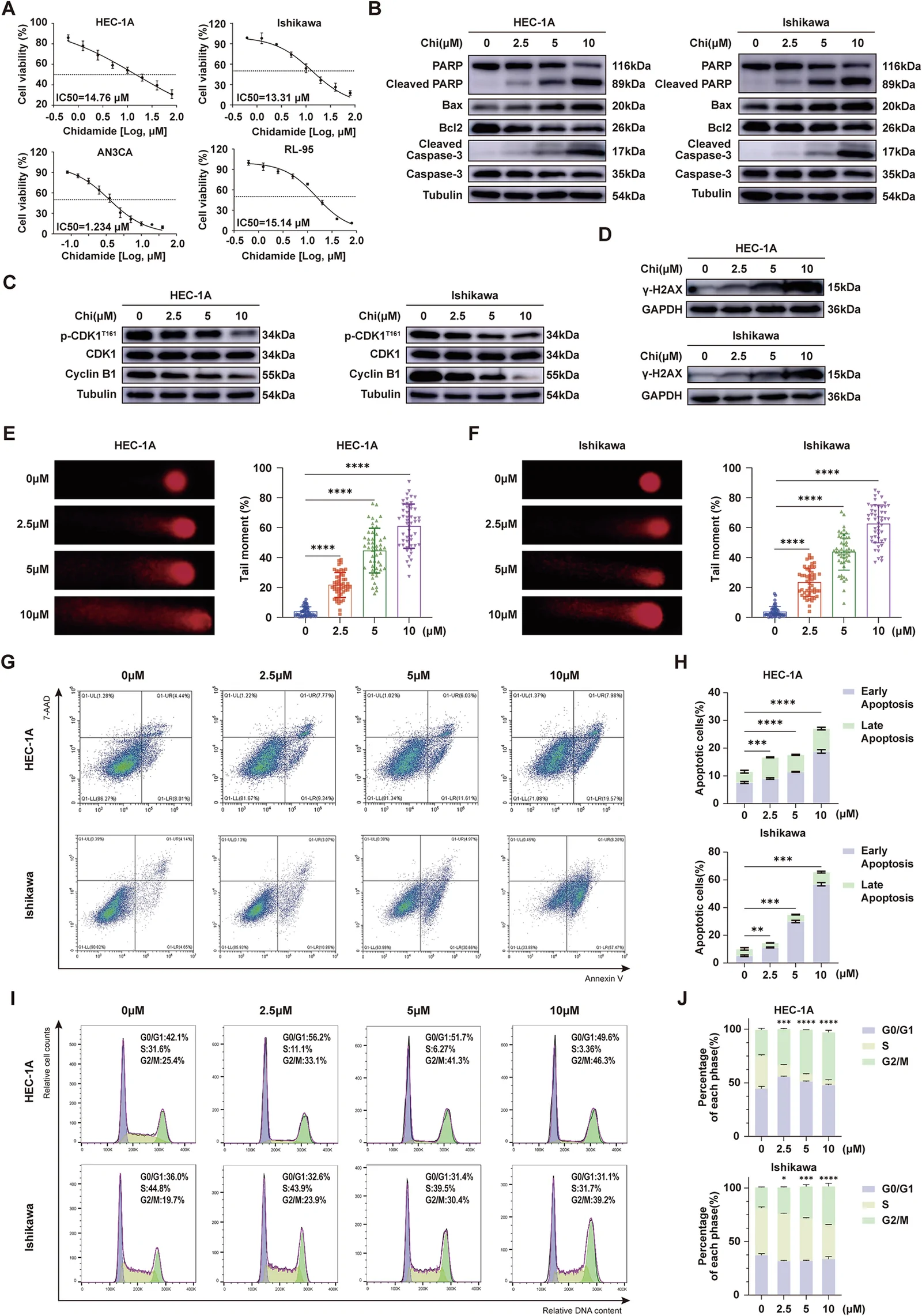
Chidamide induces cytotoxic stress, G2/M arrest, and apoptosis in endometrial cancer cells. **A** Determination of the IC50 values of chidamide in HEC-1A, Ishikawa, AN3CA, and RL95-2 cell lines. **B** Apoptosis-related markers were detected by Western blot following treatment with chidamide. **C** Cell cycle-related markers were detected by Western blot following treatment with chidamide. **D** Western blot detected γ-H2AX expression in cells following treatment with chidamide. **E** The comet assay was performed to detect DNA damage in HEC-1A cell line with quantitative analysis (*n* = 50 in each group). **F** Ishikawa cells underwent the same treatment as (**E**) for comet experiment (*n* = 50 in each group). **G** Flow cytometric analysis of apoptosis in HEC-1A and Ishikawa cells following chidamide treatment. **H** Statistical quantification of apoptotic cell percentages derived from (**G**) (*n* = 3 in each group). **I** Flow cytometric analysis of cell-cycle distribution in HEC-1A and Ishikawa cells after chidamide treatment. **J** Quantification of the proportions of cells in different phases corresponding to (**I**) (*n* = 3 in each group). ns no significance, *p < 0.05, **p < 0.01, ***p < 0.001, ****p < 0.0001.

Chidamide reduced cell viability in a dose-dependent manner after 48 h of treatment at the indicated concentrations, with IC50 values of 1.234 μM, 14.76 μM, 13.31 μM, and 15.14 μM in AN3CA, HEC-1A, Ishikawa, and RL95-2 cells, respectively (Fig. 1A). HEC-1A and Ishikawa cells were selected for subsequent mechanistic studies because they showed moderate chidamide sensitivity and represented biologically distinct EC models, allowing stress signaling analysis while avoiding excessive acute cytotoxicity. Consistently, clonogenic growth was markedly suppressed (Fig. S1G). Chidamide also induced apoptosis and caused G2/M arrest, as demonstrated by immunoblotting of apoptosis- and cell cycle-associated markers (Figs. 1B, C and S1H, I), and by flow cytometric analysis showing increased apoptotic cell fractions and G2/M-phase accumulation (Fig. 1G–J). Chidamide increased γ-H2AX expression (Figs. 1D and S1J), DNA fragmentation (Fig. 1E, F), and these findings were consistent with the immunofluorescence results (Fig. S1K, L). Together, these data show that chidamide triggers coordinated cytotoxic stress in EC cells.

### Chidamide disrupts ribosomal homeostasis and activates ribotoxic stress signaling in endometrial cancer cells

**Fig. 2:**
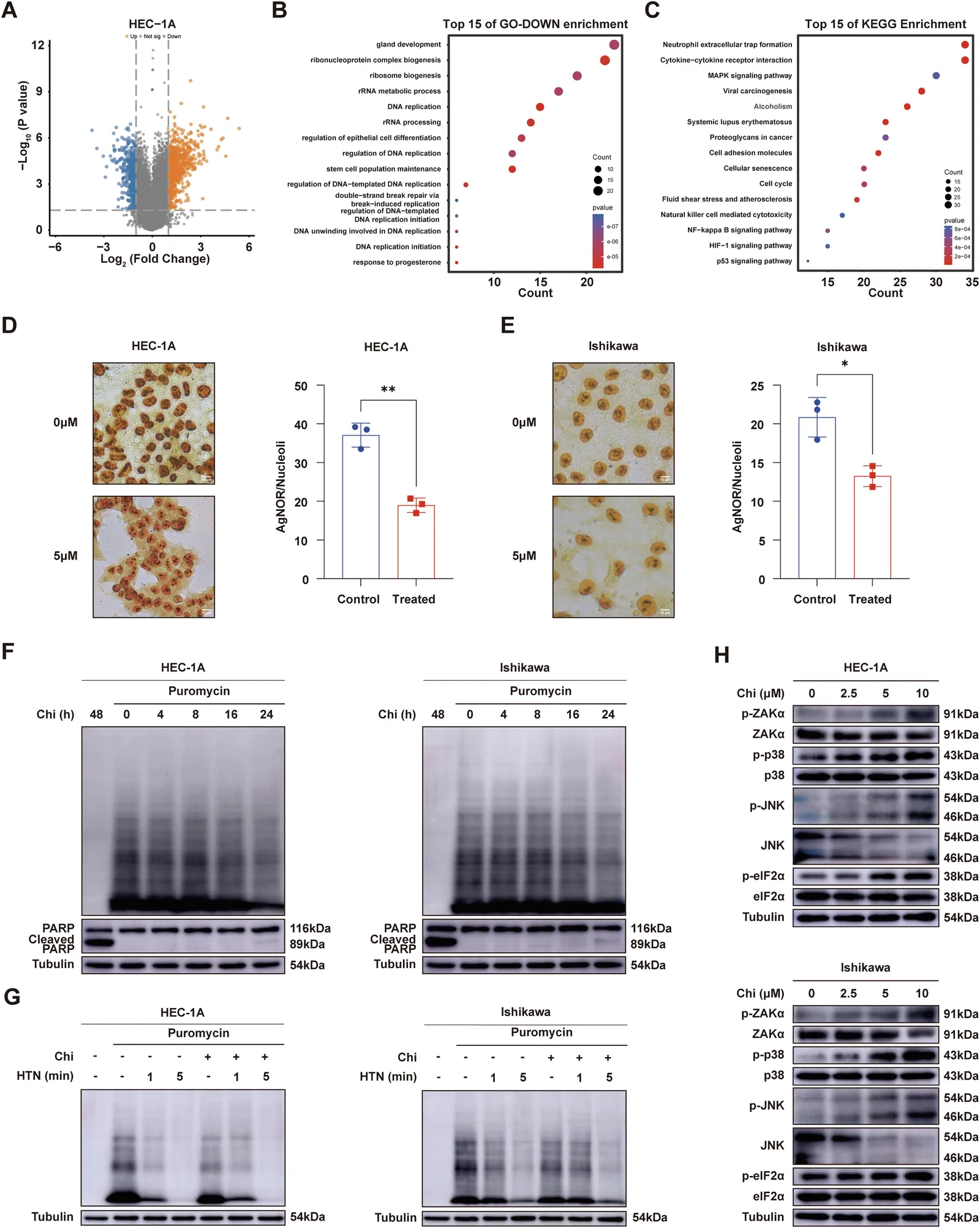
Chidamide disrupts ribosomal homeostasis and activates ribotoxic stress signaling in endometrial cancer cells. **A** Volcano plot of RNA-seq in HEC-1A cell lines. **B** Rich factor bubble plot showing top 15 terms in GO enrichment analysis of downregulated DEGs between NC and chidamide-treated groups of HEC-1A cells. **C** Rich factor bubble plot of top 15 pathways in KEGG enrichment analysis of total DEGs between NC and chidamide-treated groups HEC-1A cells. **D**, **E** Representative images and quantification of AgNOR staining in control and chidamide-treated HEC-1A and Ishikawa cells. AgNOR-positive area was normalized to nuclear area in randomly selected fields per group from each independent experiment (*n* = 3 fields in each group). **F** Representative Western blots of SUnSET assays showing nascent protein synthesis, as assessed by puromycin incorporation, in NC and chidamide-treated EC cells. Cleaved PARP was detected in parallel to assess apoptosis-associated changes. The first lane served as both a 48 h chidamide-treated apoptosis-positive reference and a no-puromycin negative control for the SUnSET assay. **G** HEC-1A and Ishikawa cells were pretreated with chidamide as indicated, followed by 2 µg/mL HTN for the indicated times to induce ribosome run-off. Puromycin (10 µg/mL) was then added for 5 min prior to harvest (after HTN treatment), and cell lysates were subjected to immunoblotting with the indicated antibodies. **H** Cells were treated with increasing concentrations of chidamide. Lysates were analyzed by immunoblotting with the indicated antibodies. ns no significance, *p < 0.05, **p < 0.01, ***p < 0.001, ****p < 0.0001.

To define the molecular basis of chidamide activity in EC, we performed RNA sequencing using HEC-1A and Ishikawa cells treated with chidamide at 5 μM, a biologically active concentration selected to induce measurable molecular changes while minimizing secondary effects from extensive cell death. Chidamide induced broad transcriptional remodeling in both cell lines (Figs. 2A and S2A). In HEC-1A cells, GO analysis of downregulated genes revealed the prominent enrichment of ribosome-associated processes, including ribonucleoprotein complex biogenesis, ribosome biogenesis, rRNA metabolic process, and rRNA processing (Fig. 2B). Similar enrichment was not observed in Ishikawa cells (Fig. S2B), indicating heterogeneity in the transcriptional response to chidamide. We next examined whether chidamide directly impairs translation. AgNOR staining revealed reduced nucleolar activity after treatment (Fig. 2D, E). SUnSET assays consistently revealed a marked reduction in puromycin incorporation following chidamide treatment, indicating the rapid suppression of global protein synthesis (Figs. 2F and S2D). Within the first 24 h of treatment, this effect occurred without the obvious induction of cleaved PARP, suggesting that translational inhibition precedes overt apoptosis. HTN-based ribosome run-off assays further showed a slower decline in puromycin incorporation in chidamide-treated cells than in control cells (Figs. 2G and S2E), suggesting delayed ribosome run-off and impaired translational elongation.

Because ribosome dysfunction can activate stress-responsive signaling, we next analyzed downstream pathways. KEGG analysis revealed the MAPK signaling pathway among the most significantly enriched pathways in both HEC-1A and Ishikawa cells (Figs. 2C and S2C). Consistent with the activation of the RSR, chidamide increased the phosphorylation of ZAKα, p38, and JNK; the phosphorylation of eIF2α was also elevated (Figs. 2H and S2F, G). Together, these results indicate that chidamide rapidly disrupts ribosomal homeostasis at both the biogenesis and translational levels while engaging parallel ribotoxic and adaptive stress signaling programs.

### ROS accumulation drives chidamide-induced translational stress and downstream cytotoxic signaling in EC cells

**Fig. 3:**
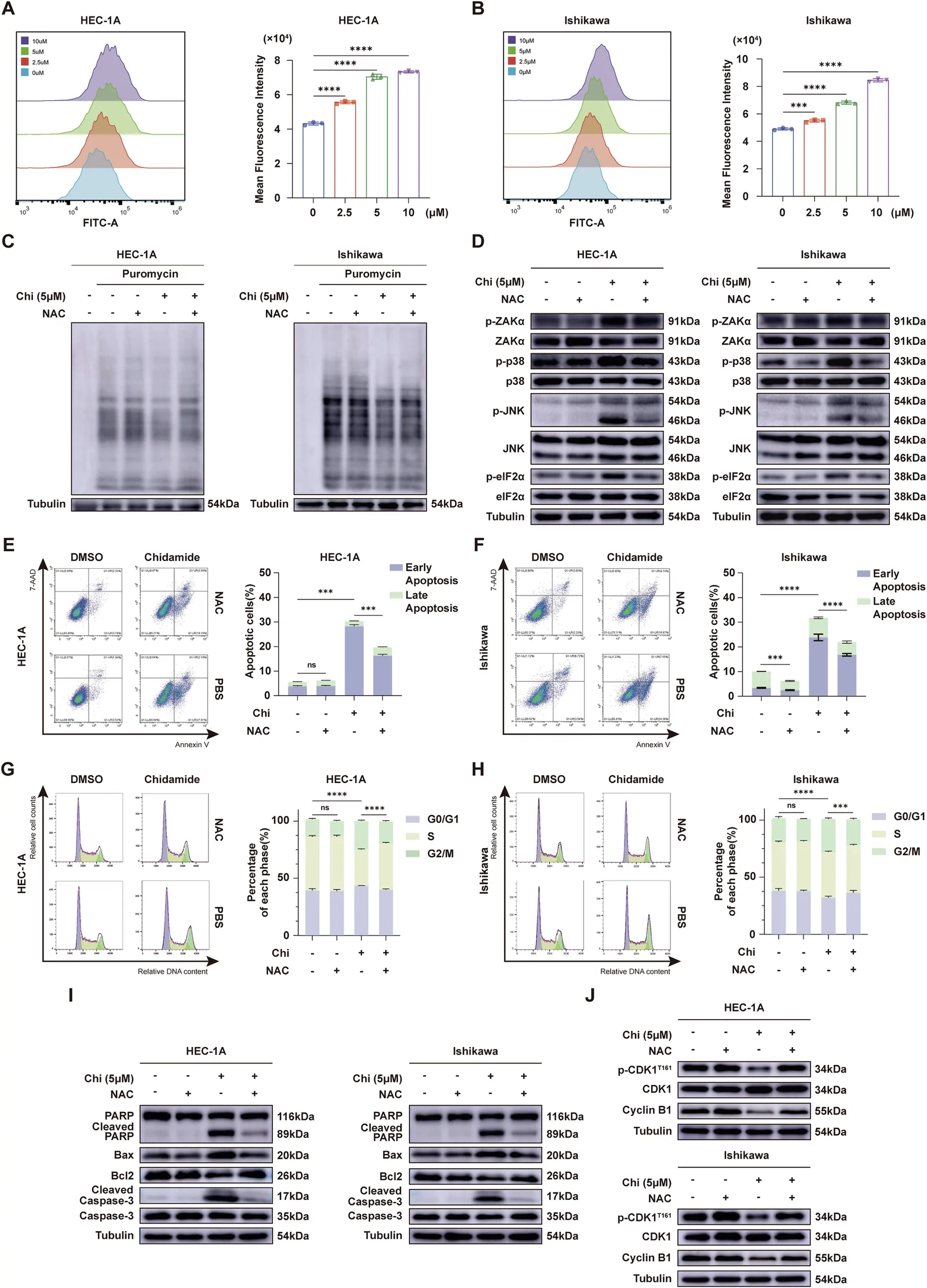
ROS accumulation drives chidamide-induced translational stress and downstream cytotoxic signaling in EC cells. **A** Detection of intracellular ROS levels of HEC-1A cells following chidamide treatment by flow cytometry and quantitative analysis (*n* = 3 in each group). **B** Ishikawa cells were treated in the same way as in (**A**) to detect intracellular ROS levels. **C** Cells were pretreated with NAC, followed by addition of chidamide as indicated. Puromycin (10 µg/mL) was added to the culture 10 min before harvest, and lysates were analyzed by immunoblotting with the antibodies of puromycin. **D** Immunoblotting of cells pretreated with NAC, followed by treatment with DMSO or chidamide, and lysates were analyzed by immunoblotting with the indicated antibodies. **E** HEC-1A cells were treated with NAC followed by treatment of chidamide. Cells were subjected to flow cytometry analysis for apoptosis detection (*n* = 3 in each group). **F** Ishikawa cells underwent the same treatment as (**E**) for apoptosis detection (*n* = 3 in each group). **G** HEC-1A cells were pretreated with NAC followed by treatment of chidamide. Following cells digestion and collection, cells were stained with PI and analyzed by flow cytometry to determine cell cycle distribution (*n* = 3 in each group). **H** Ishikawa cells were treated in the same way as in (**G**) to detect cell cycle distribution (*n* = 3 in each group). **I** Cells were pretreated with NAC, followed by addition of chidamide as indicated. Lysates were analyzed by immunoblotting with the apoptosis-associated antibodies. **J** Cells were pretreated with NAC, followed by addition of chidamide as indicated. Lysates were analyzed by immunoblotting with the cell cycle-associated antibodies. ns no significance, *p < 0.05, **p < 0.01, ***p < 0.001, ****p < 0.0001.

Given that chidamide disrupted translational homeostasis and activated ribotoxic stress signaling, we next examined whether reactive oxygen species (ROS) act upstream of this response. Flow cytometric analysis using the probe revealed that chidamide increased intracellular ROS levels in both HEC-1A and Ishikawa cells (Fig. 3A, B). To determine whether translational suppression depends on ROS, cells were pretreated with the ROS scavenger 10 mM NAC for 30 min before chidamide exposure and then subjected to SUnSET analysis. NAC markedly restored puromycin incorporation (Figs. 3C and S3A), indicating that ROS contribute to chidamide-induced translational inhibition functionally. Immunoblotting analysis further revealed that chidamide increased the phosphorylation of ZAKα, p38, JNK, and eIF2α; however, pre-treatment with NAC substantially attenuated these effects (Figs. 3D and S3B, C). At the phenotypic level, NAC significantly reduced apoptosis (Fig. 3E, F), partially reversed G2/M arrest (Fig. 3G, H), decreased apoptotic marker activation (Figs. 3I and S3D, E), and partially restored p-CDK1^T161^/CDK1 and Cyclin B1 levels (Figs. 3J and S3F, G). Together, these data identify ROS accumulation as an upstream trigger linking chidamide exposure to translational stress, stress signaling, apoptosis, and cell cycle arrest in EC cells.

### ZAKα is required for chidamide-induced stress signaling, apoptosis, and G2/M arrest in EC cells

**Fig. 4:**
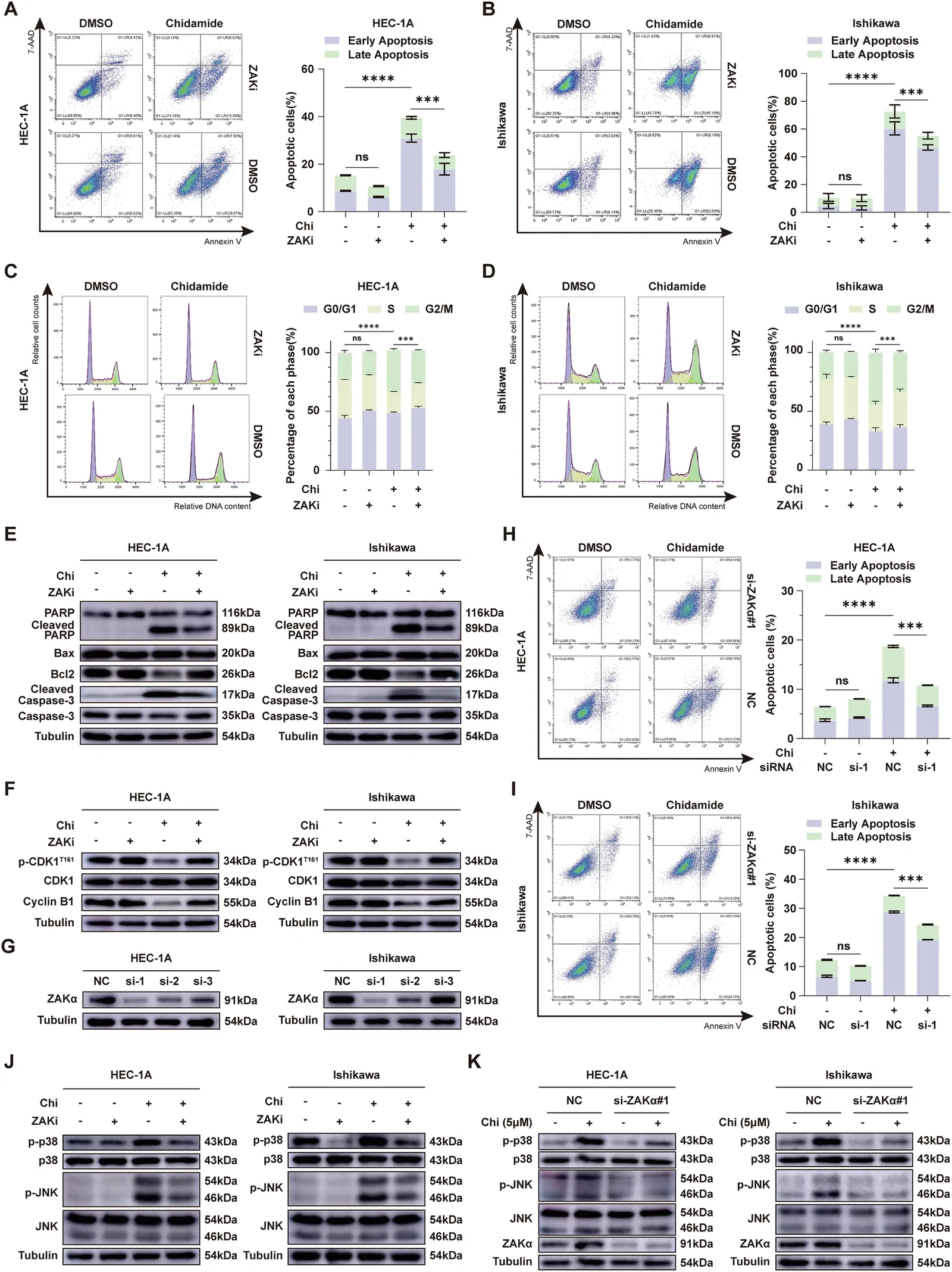
ZAKα is required for chidamide-induced stress signaling, apoptosis, and G2/M arrest in EC cells. **A** HEC-1A cells were treated with ZAKi (2 μM) followed by treatment of chidamide. Cells were subjected to flow cytometry analysis for apoptosis detection (*n* = 3 in each group). **B** Ishikawa cells underwent the same treatment as Fig. 4A for apoptosis detection (*n* = 3 in each group). **C** HEC-1A cells were pretreated with ZAKi followed by treatment of chidamide, stained with PI, and analyzed by flow cytometry to determine cell cycle distribution (*n* = 3 in each group). **D** Ishikawa cells underwent the same treatment as Fig. 4C for cell cycle detection (*n* = 3 in each group). **E** Cells were pretreated with ZAKi, followed by addition of chidamide as indicated. Lysates were analyzed by immunoblotting with the apoptosis-associated antibodies. **F** Cells were pretreated with ZAKi, followed by addition of chidamide as indicated. Lysates were analyzed by immunoblotting with the cell cycle-associated antibodies. **G** Western blot was employed to verify the silencing efficiency of ZAKα following siRNA transfection in HEC-1A and Ishikawa cells. **H** NC or si-ZAKα#1 transfected HEC-1A cells were treated with DMSO and chidamide. Cells were subjected to flow cytometry analysis for apoptosis detection (*n* = 3 in each group). **I** Ishikawa cells underwent the same treatment as Fig. 4H for apoptosis detection (*n* = 3 in each group). **J** Cells were pretreated with ZAKi followed by treatment of chidamide. Lysates were analyzed by immunoblotting with the indicated antibodies. **K** NC or si-ZAKα#1 transfected cells were treated with DMSO and chidamide. Lysates were analyzed by immunoblotting with the indicated antibodies. ns no significance, *p < 0.05, **p < 0.01, ***p < 0.001, ****p < 0.0001.

To determine whether ZAK-mediated kinase activity is functionally required for chidamide-induced stress responses, we first used a previously reported selective ZAK inhibitor (ZAKi), an ATP-competitive inhibitor of ZAK kinase activity, in HEC-1A and Ishikawa cells [20]. Cells were pretreated with ZAKi at 2 μM for 30 min according to previous reports [21, 22]. ZAK inhibition significantly reduced chidamide-induced apoptosis in both cell lines (Fig. 4A, B) and markedly alleviated G2/M arrest (Fig. 4C, D). Consistent with these findings, ZAKi decreased the cleaved PARP/PARP, cleaved caspase-3/caspase-3, and Bax/Bcl-2 ratios (Figs. 4E and S4A), and also partially restored the Cyclin B1 and p-CDK1^T161^ levels (Figs. 4F and S4B), indicating that ZAK inhibition attenuated both apoptosis and cell cycle arrest.

To exclude potential off-target effects of pharmacological inhibition, we next performed genetic validation using siRNA-mediated ZAKα knockdown. Among the three candidate siRNAs, si-ZAKα#1 and si-ZAKα#2 showed the highest knockdown efficiencies and were selected for subsequent experiments (Figs. 4G and S4C). Consistent with the effects of ZAKi treatment, transient knockdown of ZAKα using si-ZAKα#1 significantly attenuated chidamide-induced apoptosis in both HEC-1A and Ishikawa cells (Fig. 4H, I). And this effect was further validated with an independent siRNA, si-ZAKα#2, in the supplementary experiments (Fig. S4D). We then examined whether ZAKα is required for downstream stress signaling. Immunoblotting analysis showed that pharmacological ZAK inhibition and transient ZAKα knockdown using two independent siRNAs markedly attenuated chidamide-induced phosphorylation of p38 and JNK (Figs. 4J, K and S4E–H). Together, these pharmacological and genetic loss-of-function data support ZAKα as a key upstream mediator linking chidamide-induced stress signaling to p38/JNK activation, thereby contributing to apoptosis and G2/M arrest in EC cells.

### Chidamide activates a GCN2–eIF2α-dependent adaptive stress response that restrains ribotoxic signaling in EC cells

**Fig. 5:**
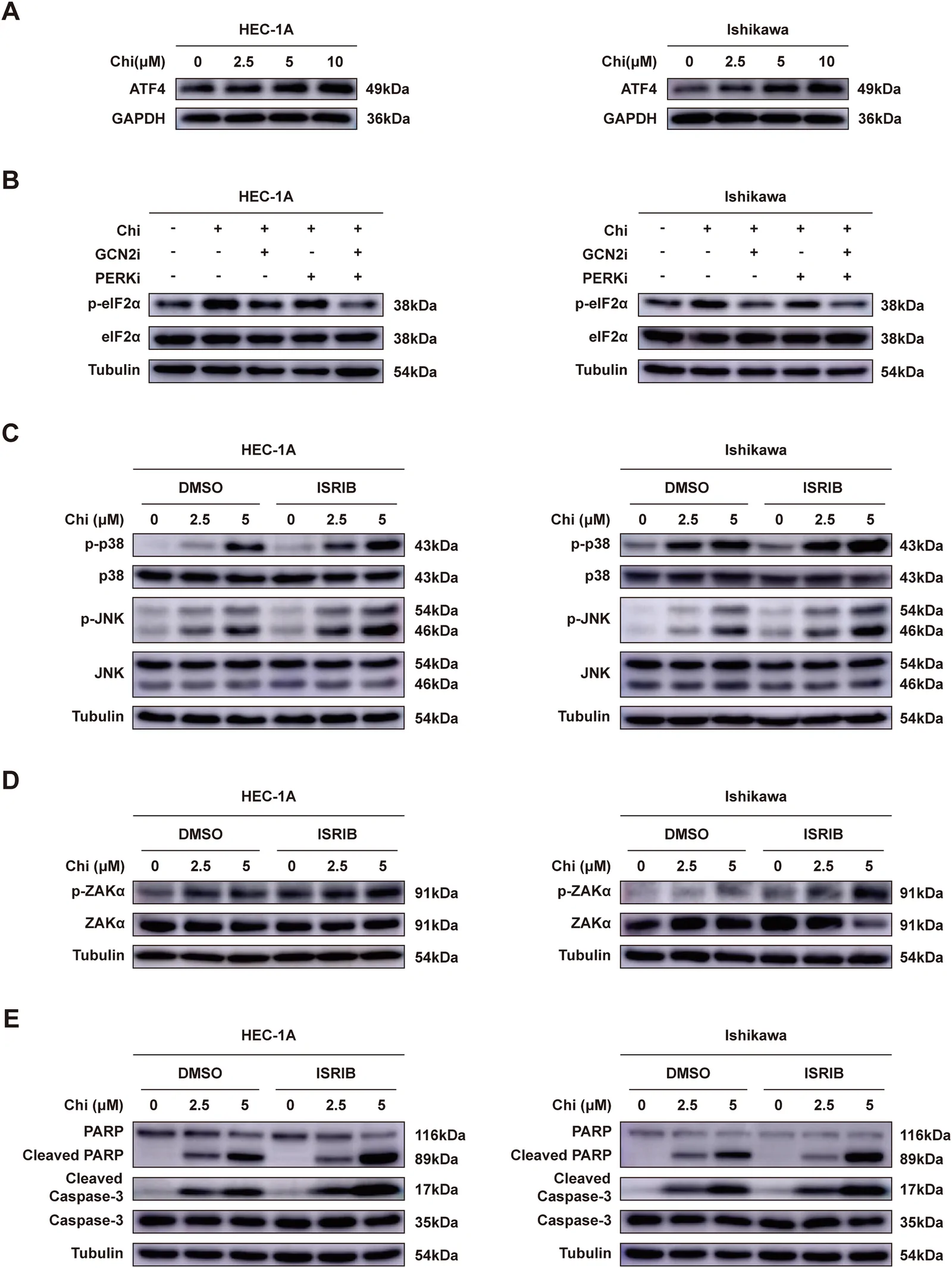
Chidamide activates a GCN2–eIF2α-dependent adaptive stress response that restrains ribotoxic signaling in EC cells. **A** Western blot analysis of ATF4 expression in EC cells following chidamide treatment. **B** Cells were pretreated with 1 μM GCN2 inhibitor and/or PERK inhibitor, followed by treatment with chidamide. Lysates were analyzed by immunoblotting with the indicated antibodies. **C** p38 and JNK phosphorylation in HEC-1A and Ishikawa cells grown in chidamide media pretreated with DMSO or ISRIB (1 μM). **D** Immunoblotting of ZAKα phosphorylation in HEC-1A and Ishikawa cells grown in media containing 0, 2.5, or 5 μM chidamide pretreated with DMSO or ISRIB. **E** Apoptosis-associated proteins in HEC-1A and Ishikawa cells grown in chidamide media pretreated with DMSO or ISRIB.

Given that chidamide can disrupt translational homeostasis and activate the ROS-dependent ribotoxic stress signaling pathway, we explored whether the adaptive translational stress program begins at the same time under these conditions. In addition to activating the RSR, chidamide also significantly increased the phosphorylation level of eIF2α in HEC-1A and Ishikawa cells, a hallmark event of ISR activation (Fig. 2H); NAC markedly weakened this effect (Fig. 3D), indicating that oxidative stress contributes to chidamide-induced ISR activation. To further determine whether the downstream pathway of the ISR was activated, we examined the expression of ATF4, a canonical downstream effector of eIF2α phosphorylation. Chidamide treatment increased ATF4 expression in EC cells (Figs. 5A and S5A), supporting activation of the eIF2α–ATF4 ISR program rather than isolated eIF2α phosphorylation.

To identify the upstream kinase responsible for eIF2α phosphorylation, we pharmacologically inhibited GCN2 or PERK using GCN2 inhibitor (A-92, 1 μM, 30 min pre-treatment) and PERK inhibitor (GSK2606414, 1 μM, 30 min pre-treatment), respectively. The inhibition of GCN2 but not PERK effectively blocked the phosphorylation of eIF2α induced by chidamide (Figs. 5B and S5B), indicating that the ISR is mediated mainly by GCN2 in this case. These data revealed that the correlation between the ISR and translational stress induced by chidamide is stronger than that between the ISR and endoplasmic reticulum stress. To determine whether the ISR functionally modulates chidamide-induced ribotoxic stress signaling pathway, we pretreated cells with integrated stress response inhibitor (ISRIB, 1 μM, 30 min pre-treatment). ISRIB acts downstream of eIF2α phosphorylation by restoring eIF2B-mediated translation initiation, thereby counteracting ISR-associated translational repression. Chidamide dose-dependently increased the phosphorylation of p38 and JNK (Fig. 5C, lanes 1–3; Fig. S5C), and ISRIB pretreatment further enhanced this effect (Fig. 5C, lanes 4–6; Fig. S5C). ISRIB also increased the ZAKα phosphorylation induced by chidamide (Figs. 5D and S5D) and further increased the levels of cleaved PARP and cleaved caspase-3 (Figs. 5E and S5E). These results indicate that chidamide can activate the GCN2–eIF2α-dependent ISR pathway, which has an adaptive inhibitory effect, limiting ZAKα–p38/JNK signaling and weakening apoptosis. Therefore, in addition to the RSR that promotes apoptosis, chidamide can also activate a parallel protective pathway to buffer translational stress in EC cells.

### The ROS–ZAKα–MAPK axis mediates chidamide-induced growth suppression in EC PDOs

**Fig. 6:**
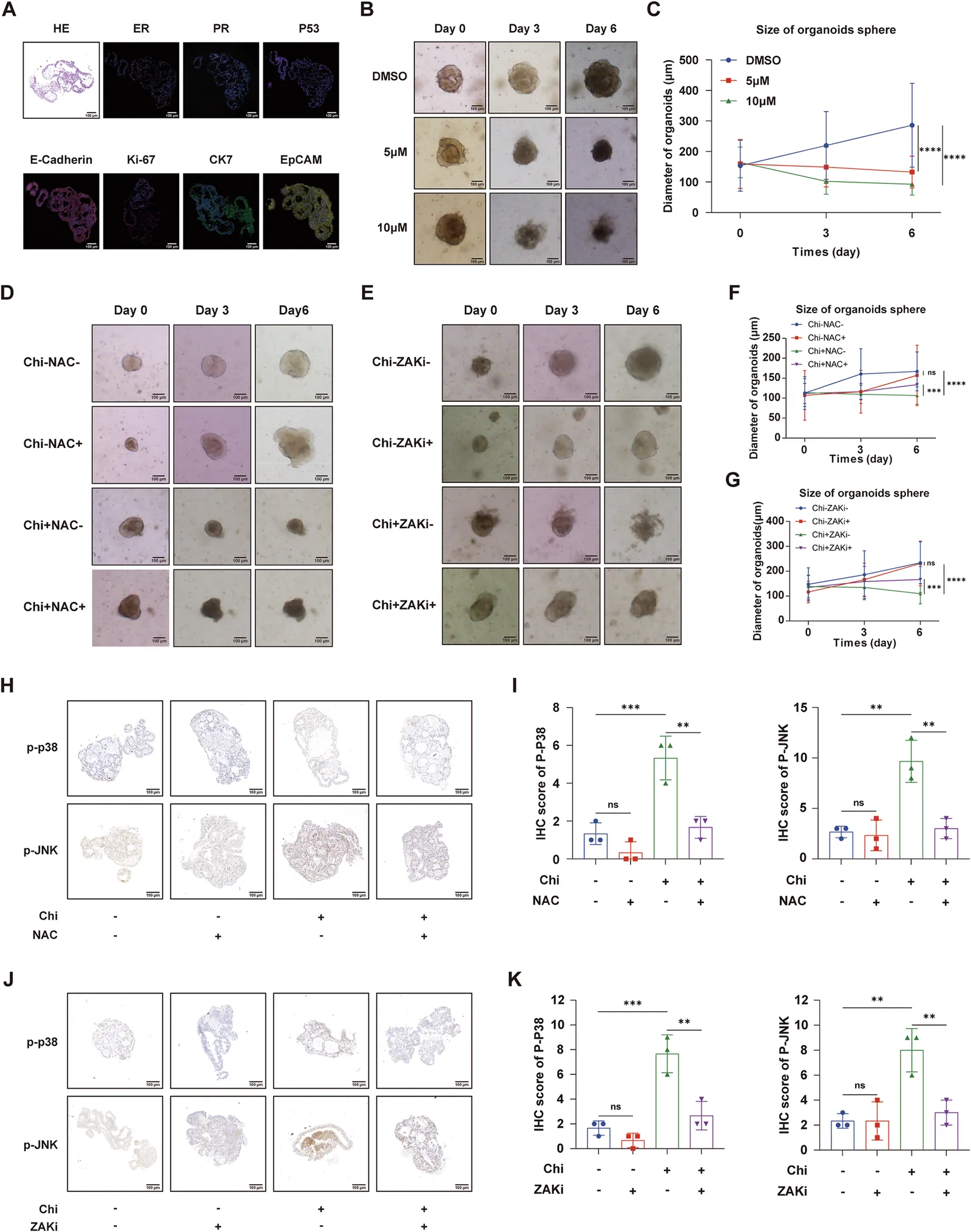
The ROS–ZAKα–MAPK axis mediates chidamide-induced growth suppression in EC PDOs. **A** Identification of organoids derived from EC tissue. **B** EC organoids were treated with chidamide at the indicated concentrations for 6 days, with DMSO-treated organoids used as controls. Images were captured immediately before treatment, defined as day 0, and on days 3 and 6 after treatment. **C** Growth curves plotted based on organoid diameter measurements from (**B**) (*n* = 50 in each group). **D** Following NAC pre-treatment, add 5 μM of chidamide and continue treatment for 6 days, and imaged. **E** Following ZAKi (2 μM) pre-treatment, add 5 μM of chidamide and continue treatment for 6 days, and imaged. **F** Growth curves plotted based on organoid diameter measurements from (**D**) (*n* = 30 in each group). **G** Growth curves plotted based on organoid diameter measurements from (**E**) (*n* = 30 in each group). **H** IHC detection of MAPK pathway activation in organoids cultured for 3 days with chidamide following NAC pre-treatment. **I** Statistical diagram of protein expression of p-p38 and p-JNK derived from (**H**) (*n* = 3 in each group). **J** IHC detection of MAPK pathway activation in organoids cultured for 3 days with chidamide following ZAKi pre-treatment. **K** Statistical diagram of protein expression of p-p38 and p-JNK derived from (**J**) (*n* = 3 in each group). ns no significance, *p < 0.05, **p < 0.01, ***p < 0.001, ****p < 0.0001.

To determine whether the mechanism identified in EC cell models could be recapitulated in a more clinically relevant system, we next evaluated chidamide in PDOs. PDOs were confirmed to retain epithelial and tumor-associated features by histology and marker expression (Fig. 6A). Chidamide at 5 and 10 μM, concentrations chosen on the basis of prior cell-line experiments to evaluate whether comparable biological effects could be observed in PDOs, progressively reduced organoid size over days 0, 3, and 6 after treatment (Fig. 6B, C). Notably, 5 μM chidamide produced marked growth inhibition while preserving evaluable organoid morphology, whereas 10 μM induced stronger suppression with more pronounced structural disruption, indicating graded antitumor activity in the PDO model.

To test the functional relevance of the ROS–ZAKα pathway, dissociated PDOs were analyzed in parallel using two sets of treatment groups. Because 5 μM chidamide was sufficient to induce a measurable PDO response while maintaining organoid integrity, this concentration was used for the subsequent NAC and ZAKi rescue experiments. For NAC experiments, PDOs were assigned to control, NAC alone, chidamide alone, and chidamide plus NAC groups. For ZAKi experiments, PDOs were assigned to control, ZAKi alone, chidamide alone, and chidamide plus ZAKi groups. Chidamide markedly inhibited PDO growth, whereas cotreatment with either NAC or ZAKi significantly attenuated this effect, as reflected by the partial recovery of growth curve and endpoint organoid size (Fig. 6D–G).

Consistently, IHC analysis revealed that after chidamide treatment, p-p38 and p-JNK staining increased in PDOs, whereas this staining was reduced after treatment with NAC and ZAKi (Fig. 6H–K). Together, these findings recapitulate the mechanism observed in EC cell models and show that chidamide suppresses EC PDO growth through a ROS–ZAKα–p38/JNK signaling axis.

### Chidamide enhances radiosensitivity and augments antitumor activity in EC models

**Fig. 7:**
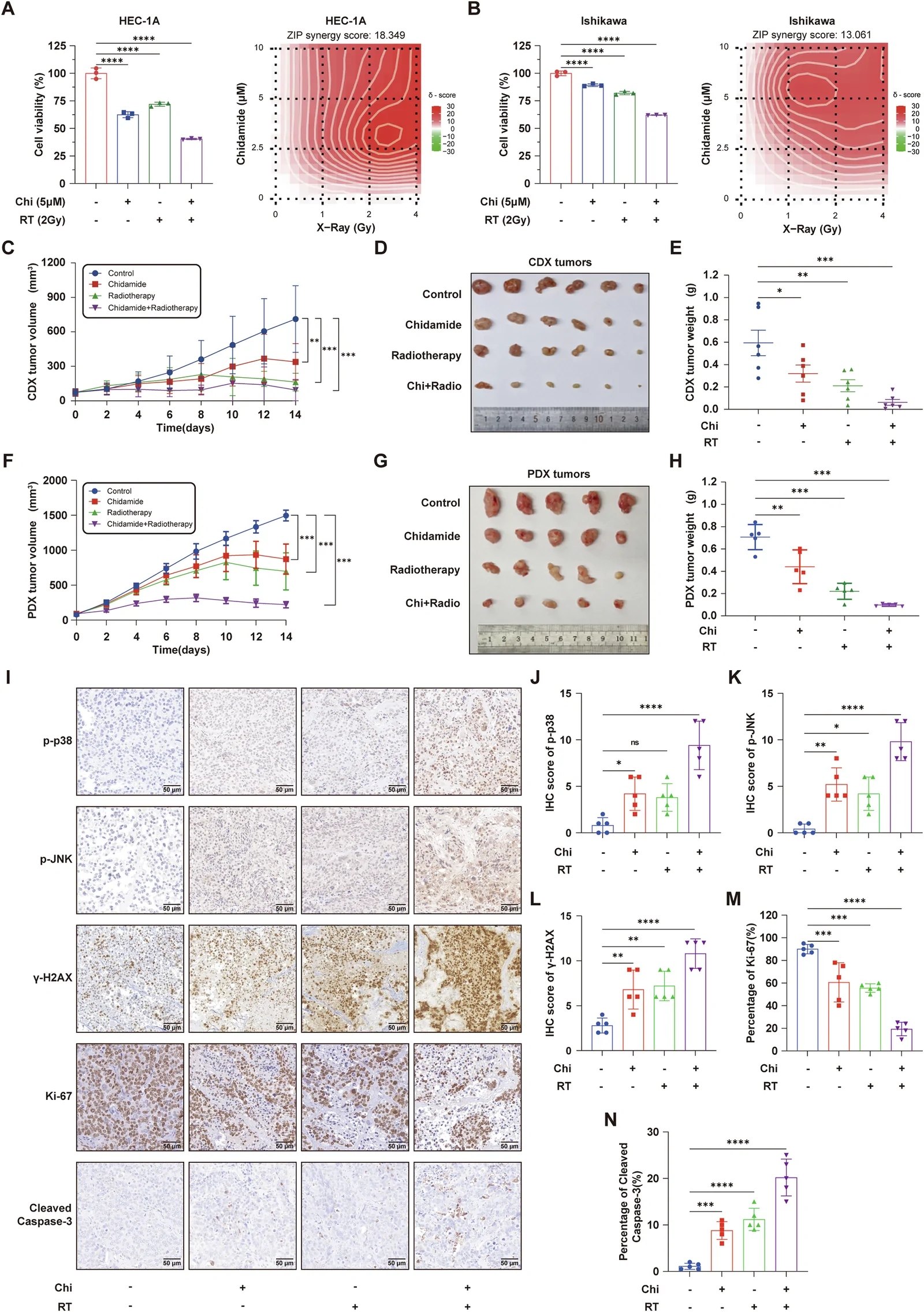
Chidamide enhances radiosensitivity and augments antitumor activity in EC models. **A** HEC-1A cell viability following treatment with chidamide or radiotherapy was determined using the CCK-8 assay and quantitatively analyzed by ZIP model (*n* = 3 in each group). **B** Ishikawa cells underwent the same treatment as (**A**) for synergy index (*n* = 3 in each group). **C**–**E** Evaluation of tumor growth in BALB/c nude mice implanted subcutaneously with 1.0 × 10⁶ HEC-1A cells and treated with chidamide or radiotherapy. **C** Tumor growth curves of CDX models. **D** Gross morphology of excised tumors. **E** Tumor weights of each treatment group (*n* = 6 in each group). **F**–**H** Tumor growth in PDX models following treatment with chidamide or radiotherapy. **F** Tumor growth curves. **G** Gross appearance of excised tumors. **H** Tumor weights of each group (*n* = 5 in each group). **I** IHC staining of indicated antibodies for effectiveness of combination therapy with chidamide and radiotherapy in PDX tumor model. **J**–**N** Quantification of indicated antibodies from (**I**) (*n* = 5 in each group). ns no significance, *p < 0.05, **p < 0.01, ***p < 0.001, ****p < 0.0001.

To evaluate the therapeutic potential of chidamide combined with radiotherapy, we evaluated its antitumor effects in vitro and in vivo. In HEC-1A and Ishikawa cells, combined treatment with chidamide and radiotherapy suppressed cell viability more effectively than either treatment alone. Quantitative analysis using the ZIP model revealed a positive synergistic effect (Fig. 7A, B), indicating that the combination therapy had a synergistic rather than simple additive inhibitory effect on EC cell growth.

Next, we studied this combination therapy in CDX and PDX models. In the CDX models established with HEC-1A cells, radiotherapy alone significantly inhibited tumor growth, and after the addition of chidamide, the tumor load further decreased, as reflected by the tumor growth curve, and reduction in terminal tumor volume and weight (Fig. 7C–E). In more clinically significant PDX models, compared with any single treatment, combined treatment had more obvious antitumor effects. Tumor growth was delayed, and the tumor volume decreased at the end point (Fig. 7F–H).

To evaluate the molecular changes related to treatment in vivo, we conducted IHC analysis of PDX tumors. Compared with those in the control group and the single treatment group, p-p38 and p-JNK staining tended to be greater in the combined treatment group, a finding that was consistent with further regulation of the stress MAPK signaling pathway in vivo (Fig. 7I–K). γ-H2AX and cleaved caspase-3 staining in the combined treatment group increased, and the expression of Ki-67 decreased (Fig. 7I, L–N), indicating that DNA damage increased, apoptosis increased, and cell proliferation decreased. In summary, these results demonstrate that chidamide can enhance the effect of radiotherapy in EC models and support its potential use in combination with radiotherapy.

## Discussion

Because the mechanism of action of chidamide in EC has remained unclear despite its known antitumor activity in other malignancies, this study aimed to define its antitumor effects in EC and elucidate the molecular basis underlying its cytotoxicity. Our research revealed that chidamide, a subtype-selective HDACi that has been confirmed to be active against hematologic malignancies, exerts marked antitumor effects in EC. In addition to the classical role of HDACis in reshaping chromatin accessibility and transcription, our data support a model in which the oxidative stress induced by chidamide disrupts translational homeostasis and activates the ZAKα-dependent RSR signaling pathway, linking ribosomal stress to downstream p38/JNK activation and cytotoxicity [23]. Previous studies have shown that HDACis can perturb redox homeostasis by impairing antioxidant defense [24, 25] and promoting mitochondrial ROS production [26]. Thus, chidamide-induced ROS accumulation in EC cells may arise from broader HDACi-associated redox dysregulation and serve as an upstream trigger for translational stress and ZAKα/MAPK activation. The pharmacological and genetic disruption of ZAKα confirms that ZAKα plays a core role in this pathway. Overall, these findings indicate that chidamide exerts antitumor activity in EC through a mechanism involving ROS-driven translational stress and activation of the ZAKα–MAPK axis, in addition to its epigenetic effects.

In addition to this central mechanism, an important conceptual development of this study is that chidamide not only activates the pro-death stress pathway but also activates adaptive responses. Like the RSR, chidamide also activated the GCN2-dependent ISR, indicating that it activates an adaptive stress program associated with translational disturbance, not classic endoplasmic reticulum stress [27]. ISRIB further enhanced stress signaling and apoptosis; this finding is consistent with the previous studies [28, 29]. The ISR acts as a protective buffer and limits the cytotoxic signaling induced by chidamide. In cancer contexts, the ISR often functions as a pro-survival mechanism, enabling tumor cells to adapt to stress and resist therapy [30, 31]. These findings support a model in which EC cells engage in two parallel but functionally different programs after being stimulated by chidamide: ZAKα-dependent RSR, which can amplify cytotoxicity signals, and GCN2-dependent ISR, which can limit the spread of stress. Therefore, the balance between these pathways may be an important determinant of therapeutic sensitivity and may help explain the differences in cellular responses. In line with this interpretation, basal class I HDAC expression alone may not fully explain the differential sensitivity of EC cell lines to chidamide, suggesting that context-dependent stress responses and apoptotic susceptibility may also influence drug sensitivity.

Consistent with this interpretation, our research revealed that ribosomal stress and translational stress are the core effects of chidamide in EC. Although HDACs are often regarded as epigenetic regulators [32], there is growing evidence that their downstream effects may converge in a broader network of stress adaptation [33, 34]. Rather than concluding that chidamide directly damages ribosomes, our findings support a model in which oxidative stress converges on ribosomal function [35], leading to translational disturbances and activating ribosome-surveillance pathways, consistent with previous findings [21]. Our results further support that stress signaling centered on ribosomes can serve as an important interface in cell fate control in cancer cells.

Importantly, these mechanistic findings from the present study were verified not only in traditional EC cell models but also in PDOs and xenograft models, supporting its biological robustness and translational significance. In PDOs, NAC and ZAKi rescue experiments supported the functional involvement of ROS accumulation and ZAKα signaling in chidamide-induced growth suppression. Xenograft models further confirmed the in vivo antitumor efficacy of chidamide and showed increased p38/JNK phosphorylation in tumor tissues, although whether this MAPK activation is directly mediated by the ROS–ZAKα axis in vivo remains to be further validated. In addition, chidamide enhanced the antitumor effect of radiotherapy both in vitro and in vivo. This radiosensitization was associated with increased DNA damage, reduced proliferation, and increased apoptosis signals in tumor tissue, indicating that chidamide may enhance the radiation response by amplifying stress-related damage signals and limiting the recovery of tumor cells [36, 37].

The enhanced antitumor effect observed with chidamide plus radiotherapy may be partly explained by the convergence of radiation-induced DNA damage and oxidative stress with chidamide-induced ROS–ZAKα signaling. Ionizing radiation can directly induce DNA damage [38] and also generate ROS through water radiolysis and radiation-associated metabolic oxidative stress [39], while ROS are closely involved in DNA damage responses induced by genotoxic therapies [40]. Recent evidence further shows that DNA damage can trigger p53-independent apoptosis through ribosome stalling, supporting a functional link between genotoxic stress and ribosome-associated stress signaling [41]. Thus, radiotherapy-induced DNA damage and oxidative stress may cooperate with chidamide-induced translational disturbance and ZAKα-dependent p38/JNK activation, thereby lowering the threshold for apoptosis. However, whether the ROS–ZAKα axis directly drives chidamide-mediated radiosensitization remains to be validated in the combined treatment setting. Given that radiotherapy is still an important part of the postoperative treatment of high-risk EC, these findings warrant further evaluation of chidamide as a potential radiosensitizer in EC treatment.

Taken together, the antitumor effects of chidamide, the involvement of the ROS–ZAKα–MAPK axis, and the adaptive buffering role of the ISR may have broader translational significance. Despite the use of multimodal treatment, high-risk EC is still associated with a high recurrence rate and poor long-term prognosis, which highlights the need for treatment strategies that address tumor-specific vulnerability. Our results show that tumors with increased oxidative stress levels, impaired translational regulation, or increased dependence on the adaptive stress buffer mechanism may be particularly sensitive to chidamide-based treatment. Although this possibility needs to be further verified, the components of the ROS–ZAKα–MAPK axis and markers of ISR activation should be explored as candidate biomarkers for response stratification. More broadly, the observation that the ISR inhibits the cytotoxicity induced by chidamide shows that the combination of chidamide with drugs that inhibit the adaptive stress response may be a reasonable strategy to improve therapeutic effects [42].

The following limitations need to be noted. EC is a heterogeneous molecular disease. We observed situation-dependent differences between different model systems, especially at the transcriptional level, which indicates that the relative contributions of the RSR and ISR may vary depending on the tumor subtype or cell state. Although our data support the central role of ZAKα, further research is needed to clarify more accurately how the oxidative stress induced by chidamide is coupled with ribosomal dysfunction at the biochemical level. Although our functional data are consistent with those of previous studies, which link translational disturbance with ZAKα-dependent ribotoxic stress signaling, we did not use higher-resolution methods such as sucrose gradient fractionation or ribosome profiling to directly evaluate ribosome distribution. Future studies using these approaches may help define how oxidative stress perturbs ribosome dynamics, clarify the precise step at which translation is impaired, and identify subtype-specific vulnerabilities that could inform patient stratification and combination therapy design.

Our study supports a mechanistic model in which chidamide induces oxidative stress-associated translational disruption, leading to activation of ZAKα-dependent MAPK signaling and concurrent engagement of the ISR. This dual stress response suggests that HDACi can engage ribosome-associated stress signaling and promote programmed cell death. These interconnected pathways appear to underlie the intrinsic cytotoxicity of chidamide and may also contribute to radiosensitization. Notably, pharmacological ISR antagonism with ISRIB partially overcomes the adaptive pro-survival buffering induced by chidamide, thereby further enhancing stress signaling and apoptosis. Together, our findings provide a rationale for further investigating chidamide in high-risk EC and support the development of rational combination strategies that exploit the balance between stress adaptation and apoptotic commitment.

## Supplementary information

Supplementary Figure Legends

Figure S1

Figure S2

Figure S3

Figure S4

Figure S5

Original Western Blots

Supplementary Tables

## Data Availability

The RNA-seq data generated in this study have been deposited in the NCBI Sequence Read Archive under BioProject accession number PRJNA1466277. The processed transcriptomic data and other data supporting the findings of this study are available from the corresponding author upon reasonable request.

## References

[1] Siegel RL, Kratzer TB, Giaquinto AN, Sung H, Jemal A. Cancer statistics, 2025. CA Cancer J Clin. 2025;75:10–45. 10.3322/caac.21871 PMC11745215 39817679

[2] Amant F, Moerman P, Neven P, Timmerman D, Van Limbergen E, Vergote I. Endometrial cancer. Lancet. 2005;366:491–505. 10.1016/S0140-6736(05)67063-8 16084259

[3] Corr BR, Erickson BK, Barber EL, Fisher CM, Slomovitz B. Advances in the management of endometrial cancer. BMJ. 2025;388:e080978. 10.1136/bmj-2024-080978 40044230

[4] Lu G, Jin S, Lin S, Gong Y, Zhang L, Yang J, et al. Update on histone deacetylase inhibitors in peripheral T-cell lymphoma (PTCL). Clin Epigenetics. 2023;15:124. 10.1186/s13148-023-01531-8 PMC10398948 37533111

[5] Jiang Z, Li W, Hu X, Zhang Q, Sun T, Cui S, et al. Tucidinostat plus exemestane for postmenopausal patients with advanced, hormone receptor-positive breast cancer (ACE): a randomised, double-blind, placebo-controlled, phase 3 trial. Lancet Oncol. 2019;20:806–15. 10.1016/S1470-2045(19)30164-0 31036468

[6] Wang F, Jin Y, Wang M, Luo HY, Fang WJ, Wang YN, et al. Combined anti-PD-1, HDAC inhibitor and anti-VEGF for MSS/pMMR colorectal cancer: a randomized phase 2 trial. Nat Med. 2024;30:1035–43. 10.1038/s41591-024-02813-1 38438735

[7] Zhang Y, Chen Z, Liu Y, Han L, Jiang W, Wang Q, et al. Chidamide plus envafolimab as subsequent treatment in advanced non-small cell lung cancer patients resistant to anti-PD-1 therapy: a multicohort, open-label, phase II trial with biomarker analysis. Cancer Med. 2024;13:e7175. 10.1002/cam4.7175 PMC11004905 38597130

[8] Cao HY, Li L, Xue SL, Dai HP. Chidamide: targeting epigenetic regulation in the treatment of hematological malignancy. Hematol Oncol. 2023;41:301–9. 10.1002/hon.3088 36251458

[9] Ning ZQ, Li ZB, Newman MJ, Shan S, Wang XH, Pan DS, et al. Chidamide (CS055/HBI-8000): a new histone deacetylase inhibitor of the benzamide class with antitumor activity and the ability to enhance immune cell-mediated tumor cell cytotoxicity. Cancer Chemother Pharmacol. 2012;69:901–9. 10.1007/s00280-011-1766-x 22080169

[10] Que Y, Zhang XL, Liu ZX, Zhao JJ, Pan QZ, Wen XZ, et al. Frequent amplification of HDAC genes and efficacy of HDAC inhibitor chidamide and PD-1 blockade combination in soft tissue sarcoma. J Immunother Cancer. 2021;9:e001696. 10.1136/jitc-2020-001696 PMC7919591 33637599

[11] Li Q, Wang F, Xiang X, Zhao L, Li X, Qu Y, et al. Chidamide and cytarabine synergistically treat acute myeloid leukemia: inhibiting ribosome biogenesis via the MYC-RRP9 pathway. Cell Death Dis. 2025;16:601. 10.1038/s41419-025-07928-y PMC12334614 40781078

[12] Sinha NK, McKenney C, Yeow ZY, Li JJ, Nam KH, Yaron-Barir TM, et al. The ribotoxic stress response drives UV-mediated cell death. Cell. 2024;187:3652–70.e40. 10.1016/j.cell.2024.05.018 PMC11246228 38843833

[13] Yan LL, Simms CL, McLoughlin F, Vierstra RD, Zaher HS. Oxidation and alkylation stresses activate ribosome-quality control. Nat Commun. 2019;10:5611. 10.1038/s41467-019-13579-3 PMC6901537 31819057

[14] Vind AC, Genzor AV, Bekker-Jensen S. Ribosomal stress-surveillance: three pathways is a magic number. Nucleic Acids Res. 2020;48:10648–61. 10.1093/nar/gkaa757 PMC7641731 32941609

[15] Taniuchi S, Miyake M, Tsugawa K, Oyadomari M, Oyadomari S. Integrated stress response of vertebrates is regulated by four eIF2α kinases. Sci Rep. 2016;6:32886. 10.1038/srep32886 PMC5025754 27633668

[16] Ishimura R, Nagy G, Dotu I, Chuang JH, Ackerman SL. Activation of GCN2 kinase by ribosome stalling links translation elongation with translation initiation. eLife. 2016;5:e14295. 10.7554/eLife.14295 PMC4917338 27085088

[17] Stoneley M, Harvey RF, Mulroney TE, Mordue R, Jukes-Jones R, Cain K, et al. Unresolved stalled ribosome complexes restrict cell-cycle progression after genotoxic stress. Mol Cell. 2022;82:1557–72.e7. 10.1016/j.molcel.2022.01.019 PMC9098122 35180429

[18] McKenney CD, Regot S. Cell cycle regulation by the ribotoxic stress response. Trends Cell Biol. 2025;35:592–603. 10.1016/j.tcb.2025.04.005 PMC12227297 40379527

[19] Dhanasekaran DN, Reddy EP. JNK signaling in apoptosis. Oncogene. 2008;27:6245–51. 10.1038/onc.2008.301 PMC3063296 18931691

[20] Chang Y, Lu X, Shibu MA, Dai Y-B, Luo J, Zhang Y, et al. Structure Based Design of N-(3-((1H-Pyrazolo[3,4-b]pyridin-5-yl)ethynyl)benzenesulfonamides as Selective Leucine-Zipper and Sterile-α Motif Kinase (ZAK) Inhibitors. J Med Chem. 2017;60:5927–32. 10.1021/acs.jmedchem.7b00572 28586211

[21] Snieckute G, Ryder L, Vind AC, Wu Z, Arendrup FS, Stoneley M, et al. ROS-induced ribosome impairment underlies ZAKα-mediated metabolic decline in obesity and aging. Science. 2023;382:eadf3208. 10.1126/science.adf3208 38060659

[22] Ryder L, Arendrup FS, Martínez JF, Snieckute G, Pecorari C, Shah RA, et al. Nitric oxide-induced ribosome collision activates ribosomal surveillance mechanisms. Cell Death Dis. 2023;14:467. 10.1038/s41419-023-05997-5 PMC10372077 37495584

[23] Wu CC, Peterson A, Zinshteyn B, Regot S, Green R. Ribosome collisions trigger general stress responses to regulate cell fate. Cell. 2020;182:404–16.e14. 10.1016/j.cell.2020.06.006 PMC7384957 32610081

[24] Butler LM, Zhou X, Xu W-S, Scher HI, Rifkind RA, Marks PA, et al. The histone deacetylase inhibitor SAHA arrests cancer cell growth, up-regulates thioredoxin-binding protein-2, and down-regulates thioredoxin. Proc Natl Acad Sci USA. 2002;99:11700–5. 10.1073/pnas.182372299 PMC129332 12189205

[25] Ungerstedt JS, Sowa Y, Xu WS, Shao Y, Dokmanovic M, Perez G, et al. Role of thioredoxin in the response of normal and transformed cells to histone deacetylase inhibitors. Proc Natl Acad Sci USA. 2005;102:673–8. 10.1073/pnas.0408732102 PMC543461 15637150

[26] Rosato RR, Almenara JA, Grant S. The histone deacetylase inhibitor MS-275 promotes differentiation or apoptosis in human leukemia cells through a process regulated by generation of reactive oxygen species and induction of p21CIP1/WAF1 1. Cancer Res. 2003;63:3637–45. 12839953

[27] Montalbano R, Waldegger P, Quint K, Jabari S, Neureiter D, Illig R, et al. Endoplasmic reticulum stress plays a pivotal role in cell death mediated by the pan-deacetylase inhibitor panobinostat in human hepatocellular cancer cells. Transl Oncol. 2013;6:143–57. 10.1593/tlo.12271 PMC3610545 23544167

[28] Nguyen HG, Conn CS, Kye Y, Xue L, Forester CM, Cowan JE, et al. Development of a stress response therapy targeting aggressive prostate cancer. Sci Transl Med. 2018;10:eaar2036. 10.1126/scitranslmed.aar2036 PMC6045425 29720449

[29] Palam LR, Gore J, Craven KE, Wilson JL, Korc M. Integrated stress response is critical for gemcitabine resistance in pancreatic ductal adenocarcinoma. Cell Death Dis. 2015;6:e1913. 10.1038/cddis.2015.264 PMC4632294 26469962

[30] Ghaddar N, Wang S, Woodvine B, Krishnamoorthy J, van Hoef V, Darini C, et al. The integrated stress response is tumorigenic and constitutes a therapeutic liability in KRAS-driven lung cancer. Nat Commun. 2021;12:4651. 10.1038/s41467-021-24661-0 PMC8324901 34330898

[31] Lines CL, McGrath MJ, Dorwart T, Conn CS. The integrated stress response in cancer progression: a force for plasticity and resistance. Front Oncol. 2023;13:1206561. 10.3389/fonc.2023.1206561 PMC10435748 37601686

[32] Minucci S, Pelicci PG. Histone deacetylase inhibitors and the promise of epigenetic (and more) treatments for cancer. Nat Rev Cancer. 2006;6:38–51. 10.1038/nrc1779 16397526

[33] Roos WP, Krumm A. The multifaceted influence of histone deacetylases on DNA damage signalling and DNA repair. Nucleic Acids Res. 2016;44:10017–30. 10.1093/nar/gkw922 PMC5137451 27738139

[34] Wells CE, Bhaskara S, Stengel KR, Zhao Y, Sirbu B, Chagot B, et al. Inhibition of histone deacetylase 3 causes replication stress in cutaneous T cell lymphoma. PLoS ONE. 2013;8:e68915. 10.1371/journal.pone.0068915 PMC3718806 23894374

[35] Rosato RR, Almenara JA, Maggio SC, Coe S, Atadja P, Dent P, et al. Role of histone deacetylase inhibitor-induced reactive oxygen species and DNA damage in LAQ-824/fludarabine antileukemic interactions. Mol Cancer Ther. 2008;7:3285–97. 10.1158/1535-7163.MCT-08-0385 PMC2586957 18852132

[36] Groselj B, Sharma NL, Hamdy FC, Kerr M, Kiltie AE. Histone deacetylase inhibitors as radiosensitisers: effects on DNA damage signalling and repair. Br J Cancer. 2013;108:748–54. 10.1038/bjc.2013.21 PMC3590661 23361058

[37] Xia F, Ping N, Bao Q, Zou R, Zhu Q, Zhang X, et al. Addition of chidamide to a sandwich protocol, pegaspargase, gemcitabine and oxaliplatin chemotherapy combined with radiotherapy, as first-line treatment in early stage NK/T-cell lymphoma: real world outcomes in a retrospective study. Blood. 2023;142:6202.

[38] Santivasi WL, Xia F. Ionizing radiation-induced DNA damage, response, and repair. Antioxid Redox Signal. 2014;21:251–9. 10.1089/ars.2013.5668 24180216

[39] Azzam EI, Jay-Gerin J-P, Pain D. Ionizing radiation-induced metabolic oxidative stress and prolonged cell injury. Cancer Lett. 2012;327:48–60. 10.1016/j.canlet.2011.12.012 PMC3980444 22182453

[40] Srinivas US, Tan BWQ, Vellayappan BA, Jeyasekharan AD. ROS and the DNA damage response in cancer. Redox Biol. 2019;25:101084. 10.1016/j.redox.2018.101084 PMC6859528 30612957

[41] Boon NJ, Oliveira RA, Körner P-R, Kochavi A, Mertens S, Malka Y, et al. DNA damage induces p53-independent apoptosis through ribosome stalling. Science. 2024;384:785–92. 10.1126/science.adh7950 38753784

[42] Labrie M, Brugge JS, Mills GB, Zervantonakis IK. Therapy resistance: opportunities created by adaptive responses to targeted therapies in cancer. Nat Rev Cancer. 2022;22:323–39. 10.1038/s41568-022-00454-5 PMC9149051 35264777

