# Supplementary material for: Chidamide induces ROS-driven ZAKα-dependent ribotoxic stress in endometrial cancer and enhances radiosensitivity in vitro and in vivo
Source: Cell Death Dis. 2026 Jul 23;17(1):892. doi: 10.1038/s41419-026-09125-x (PMC13680392; doi:10.1038/s41419-026-09125-x)

Figure 1B

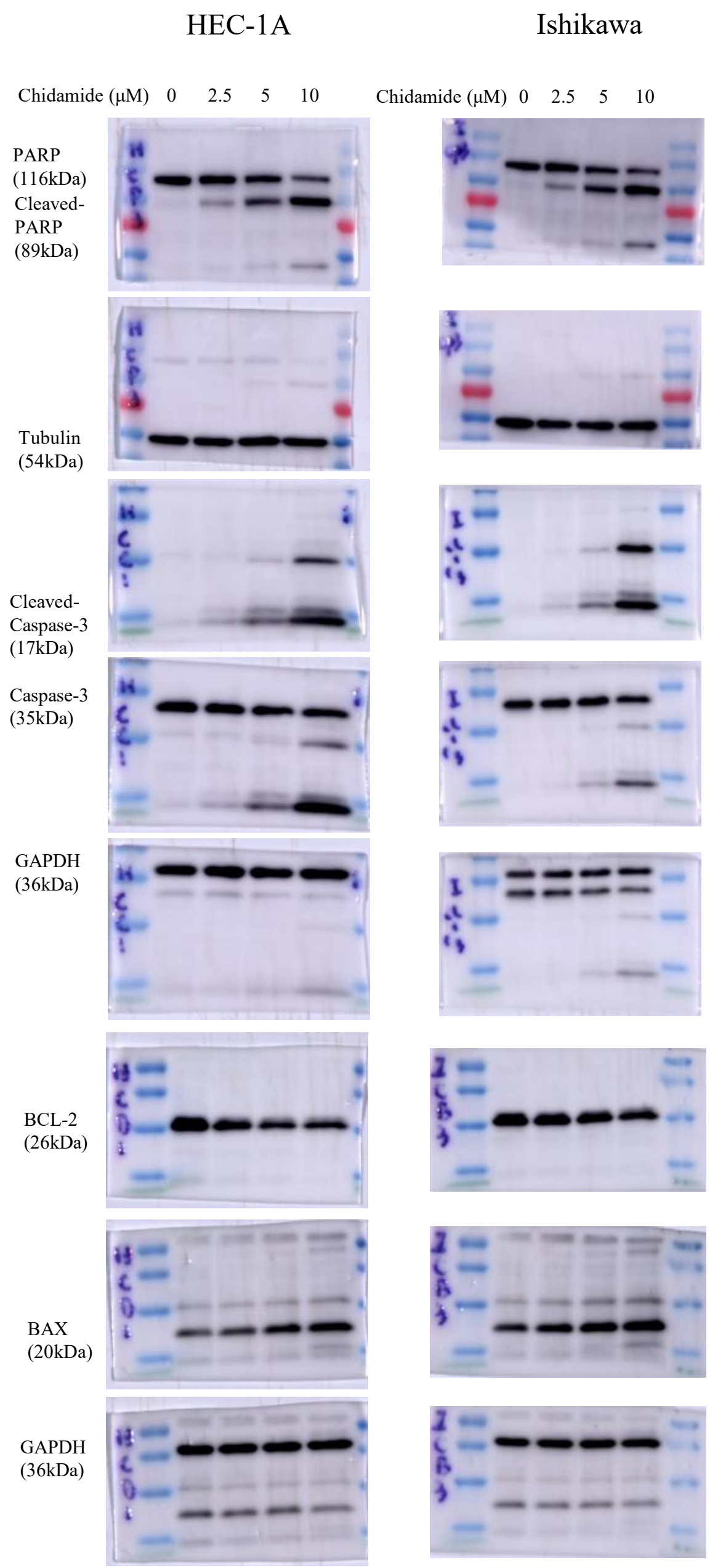

Figure 1C

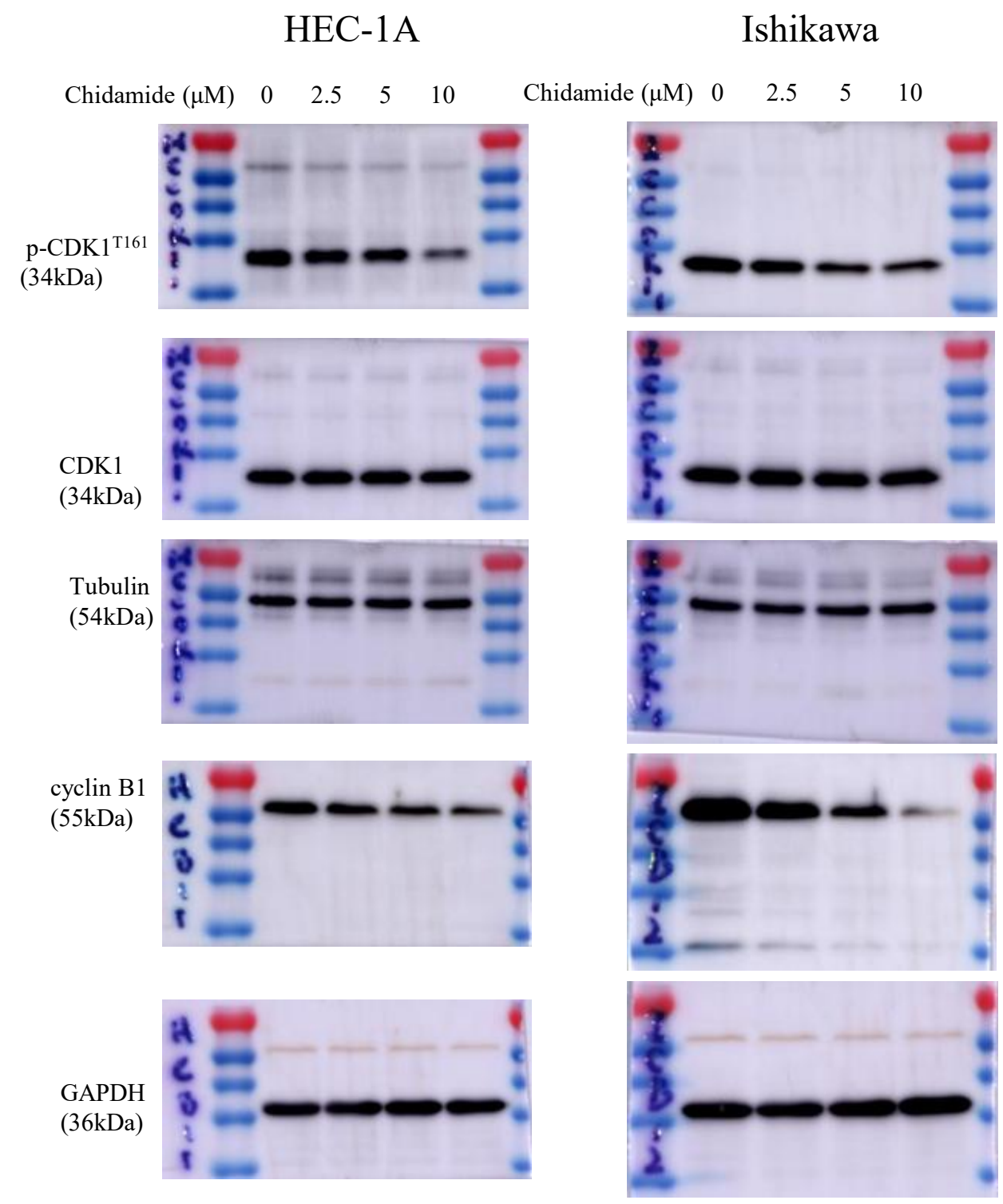

Figure 1D

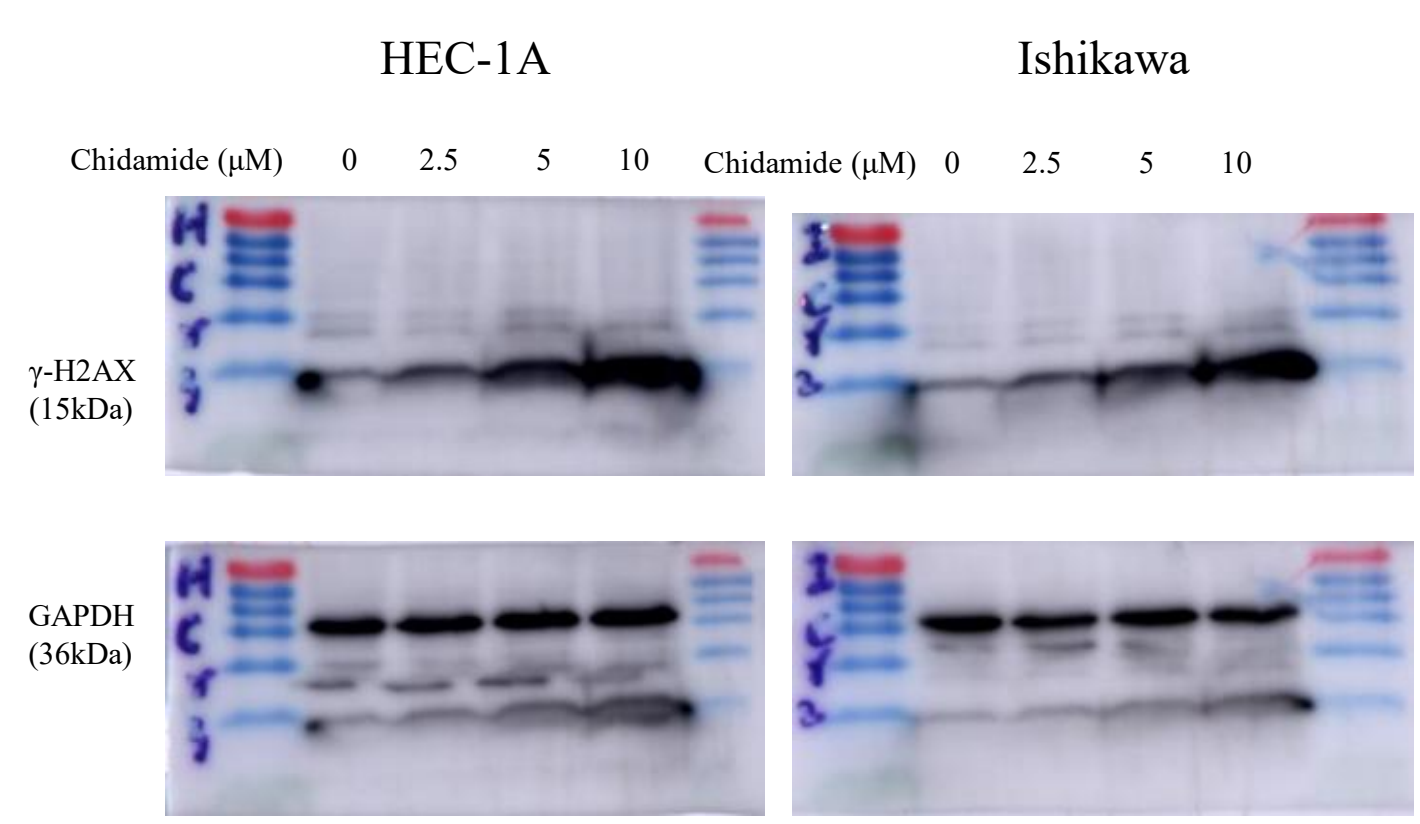

Figure 2F

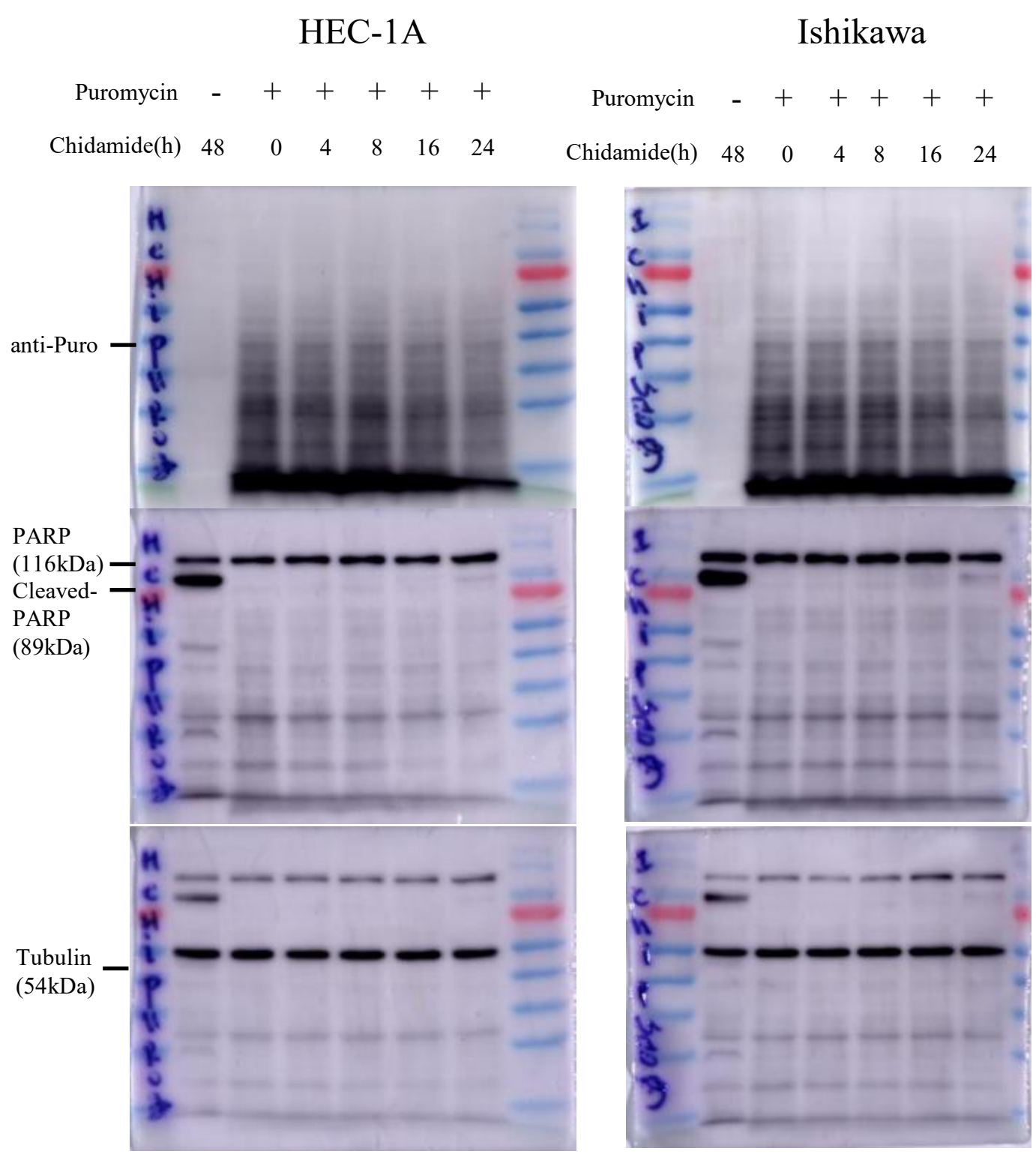

Figure 2G

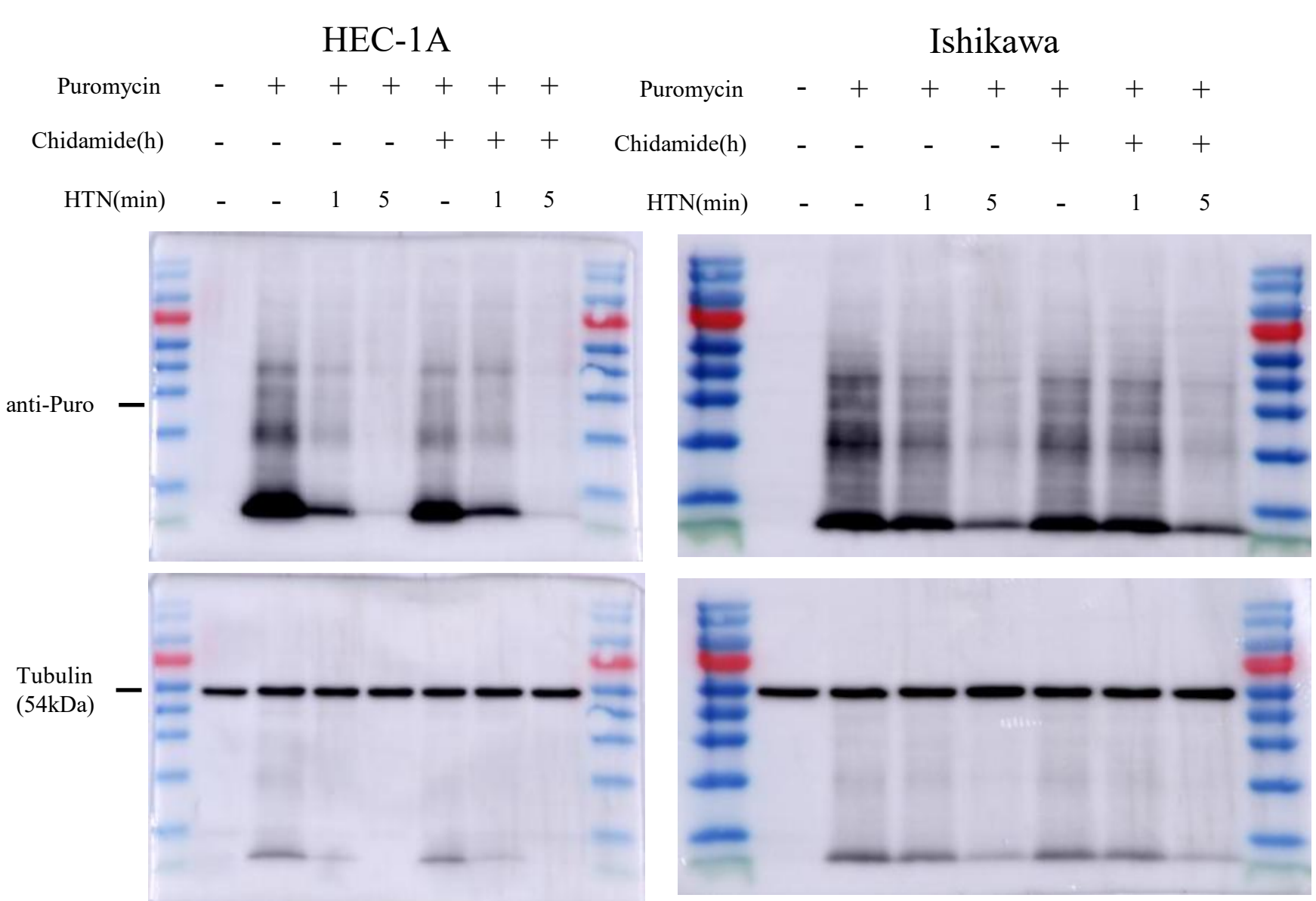

Figure 2H

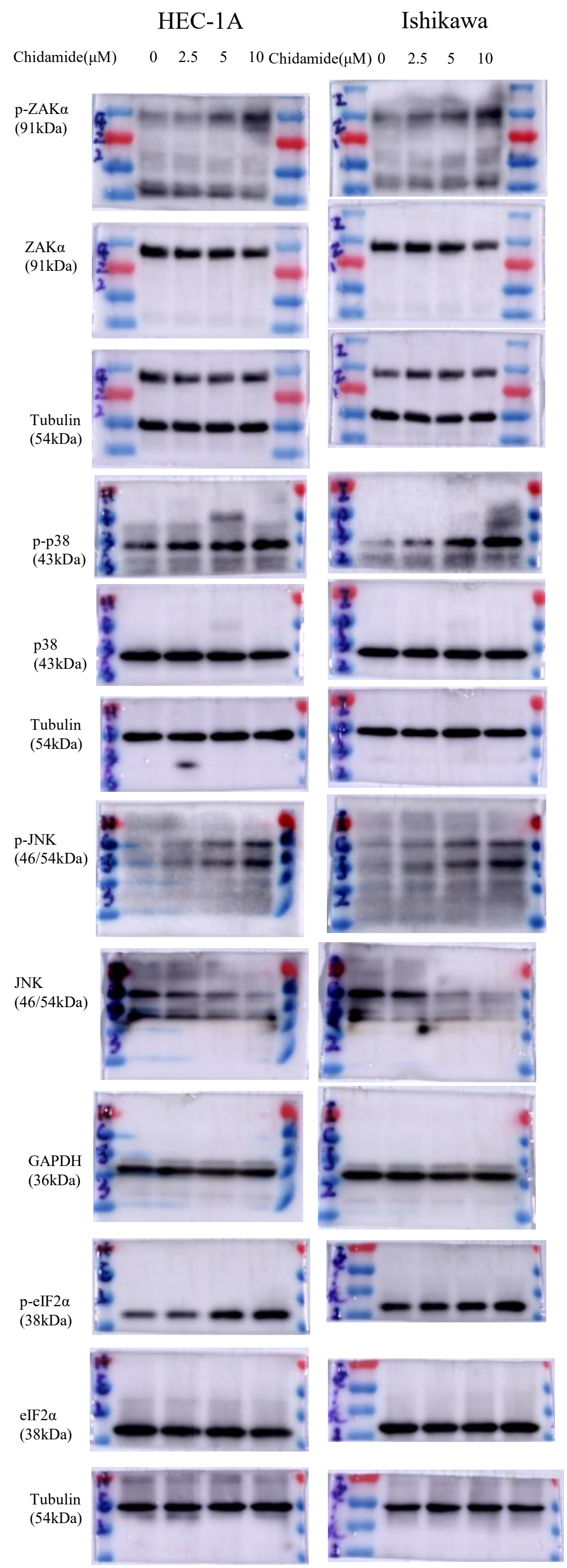

Figure 3C

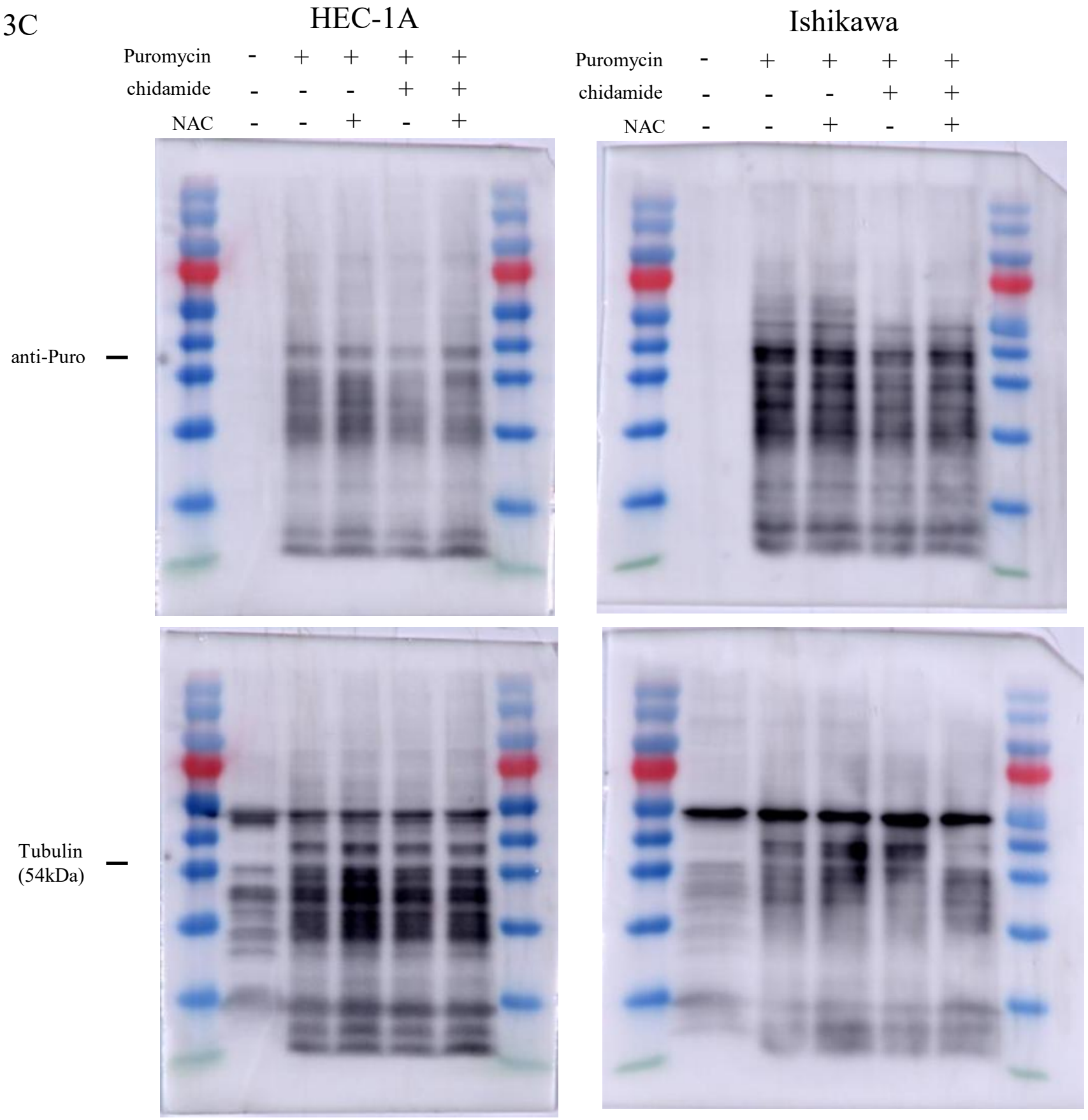

Figure 3D

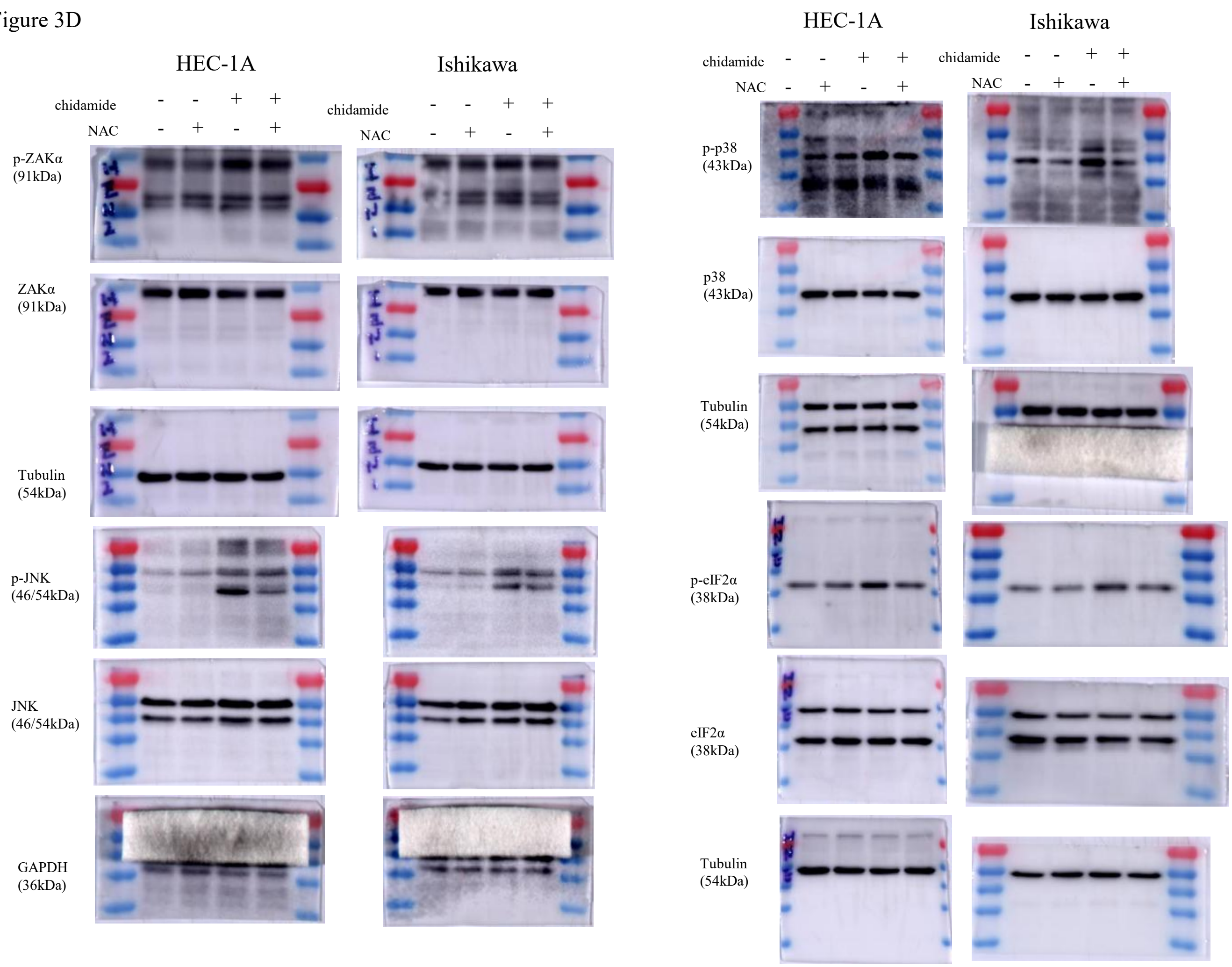

Figure 3I

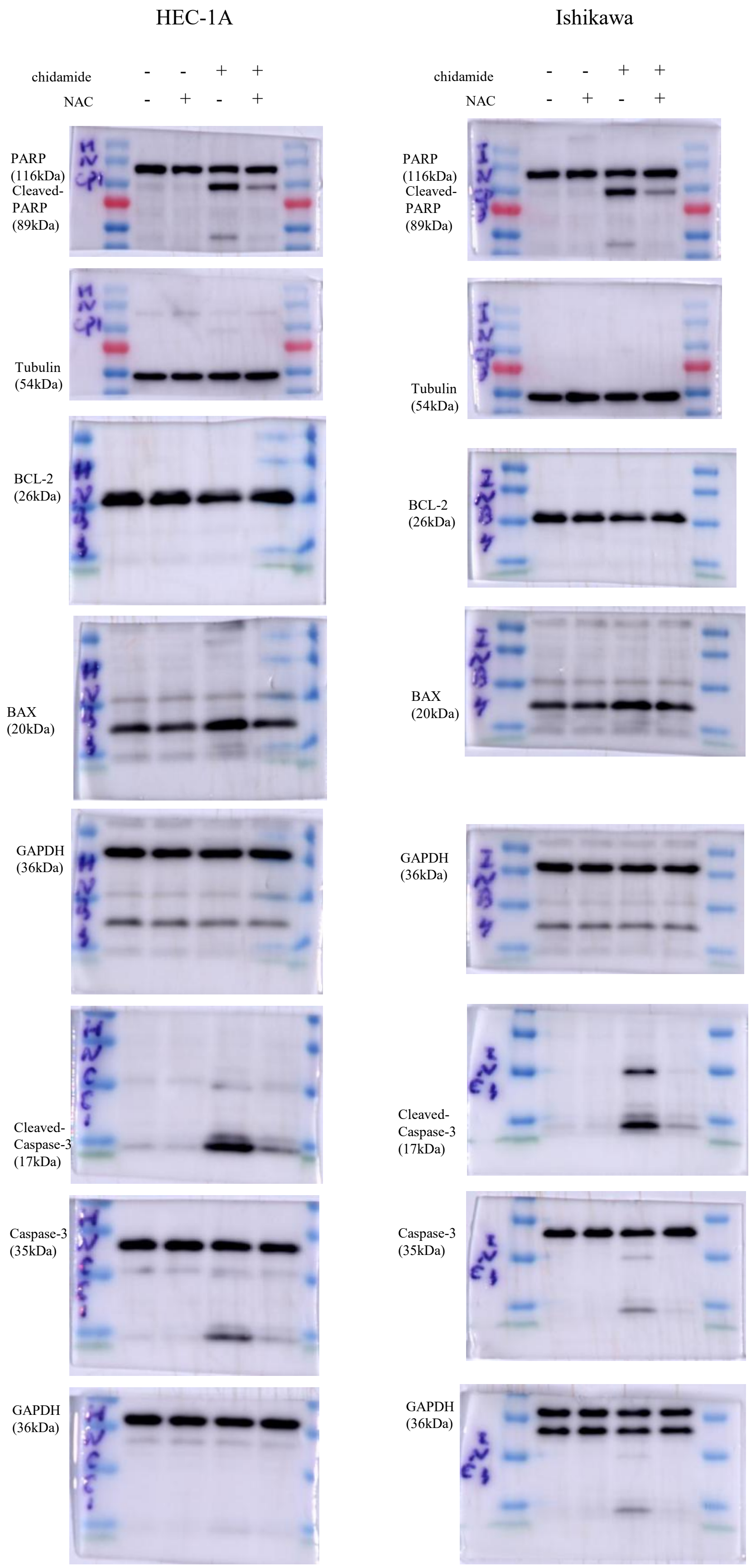

Figure 3J

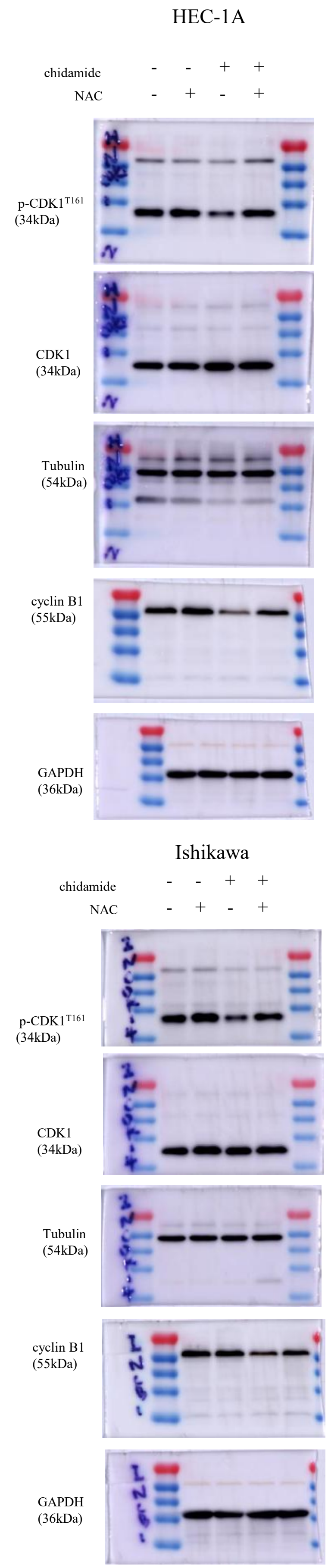

Figure 4E

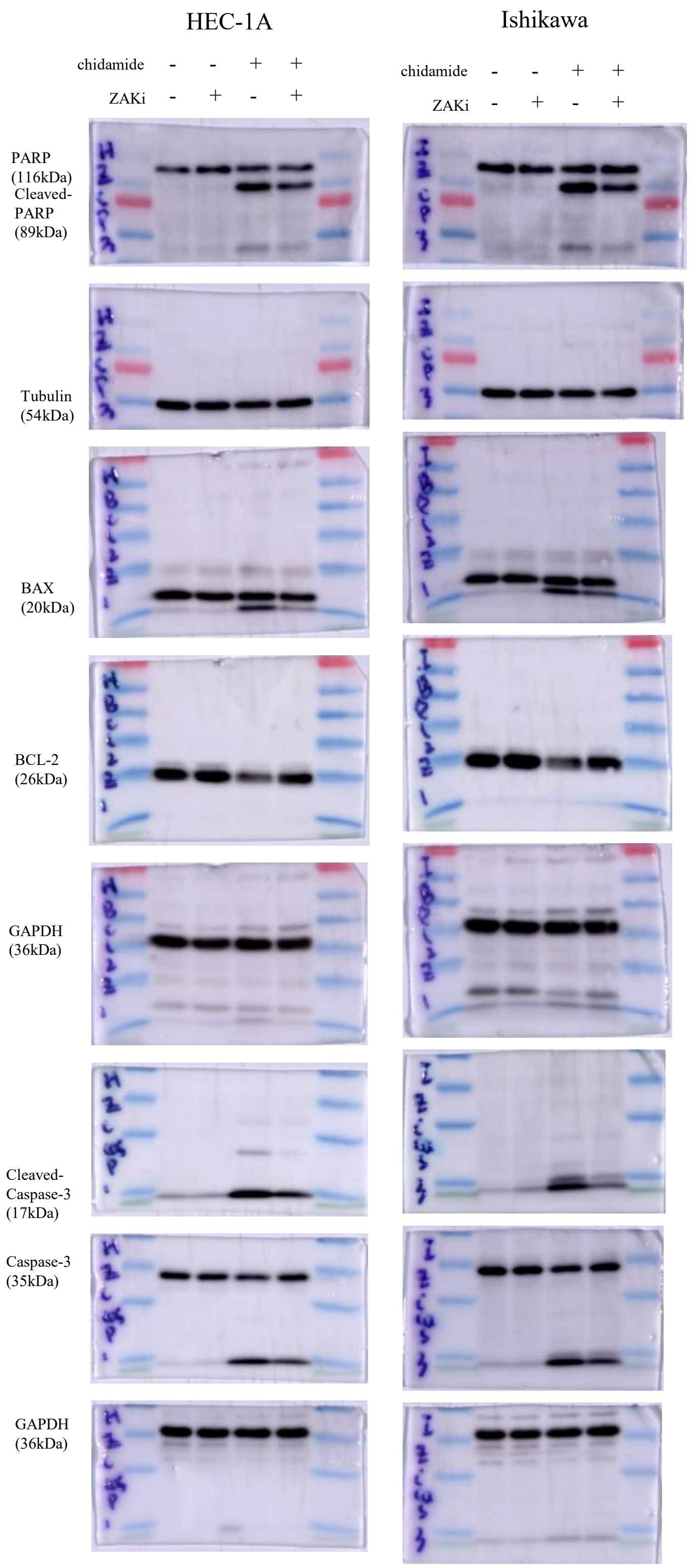

Figure 4F

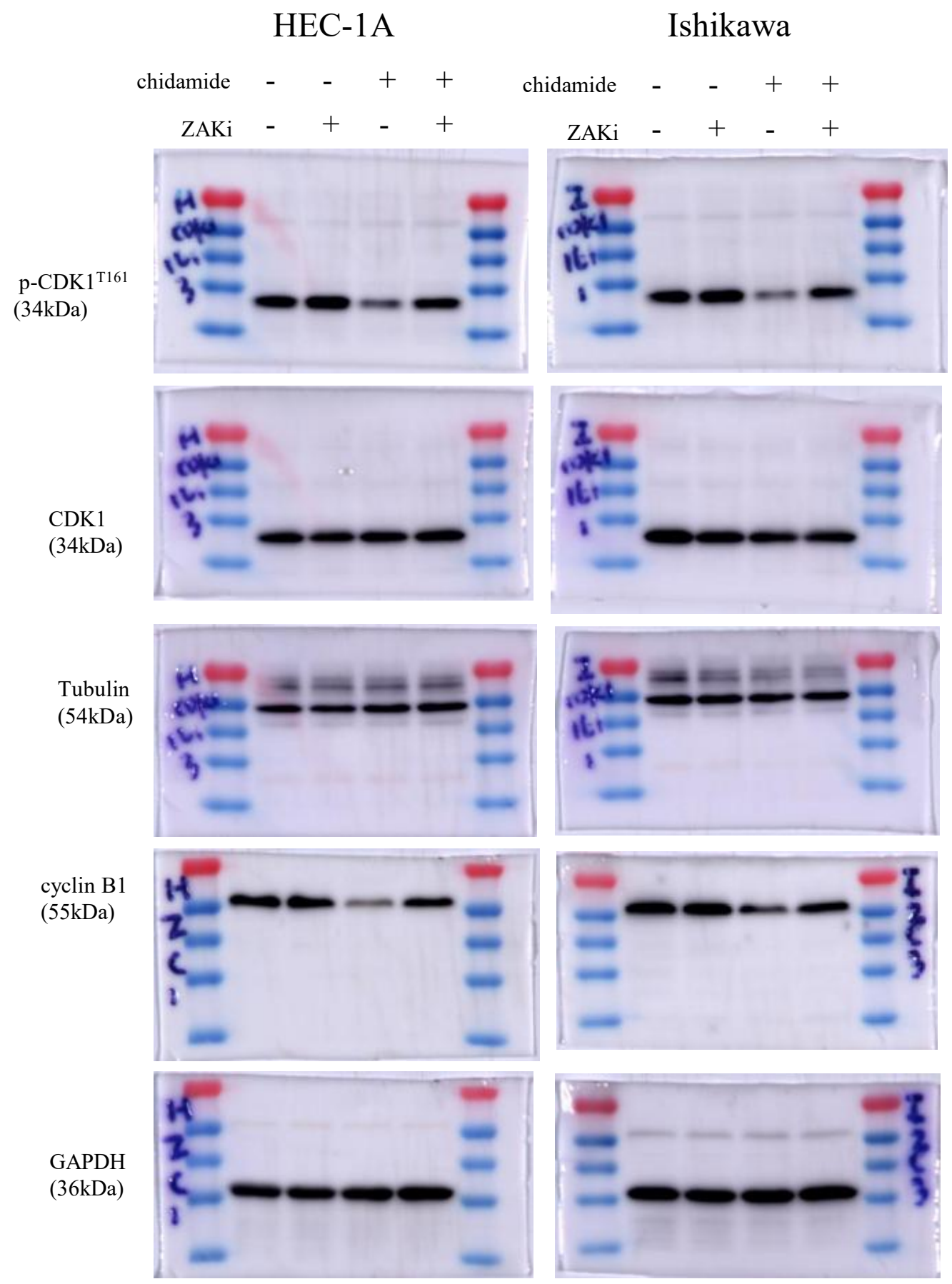

Figure 4G

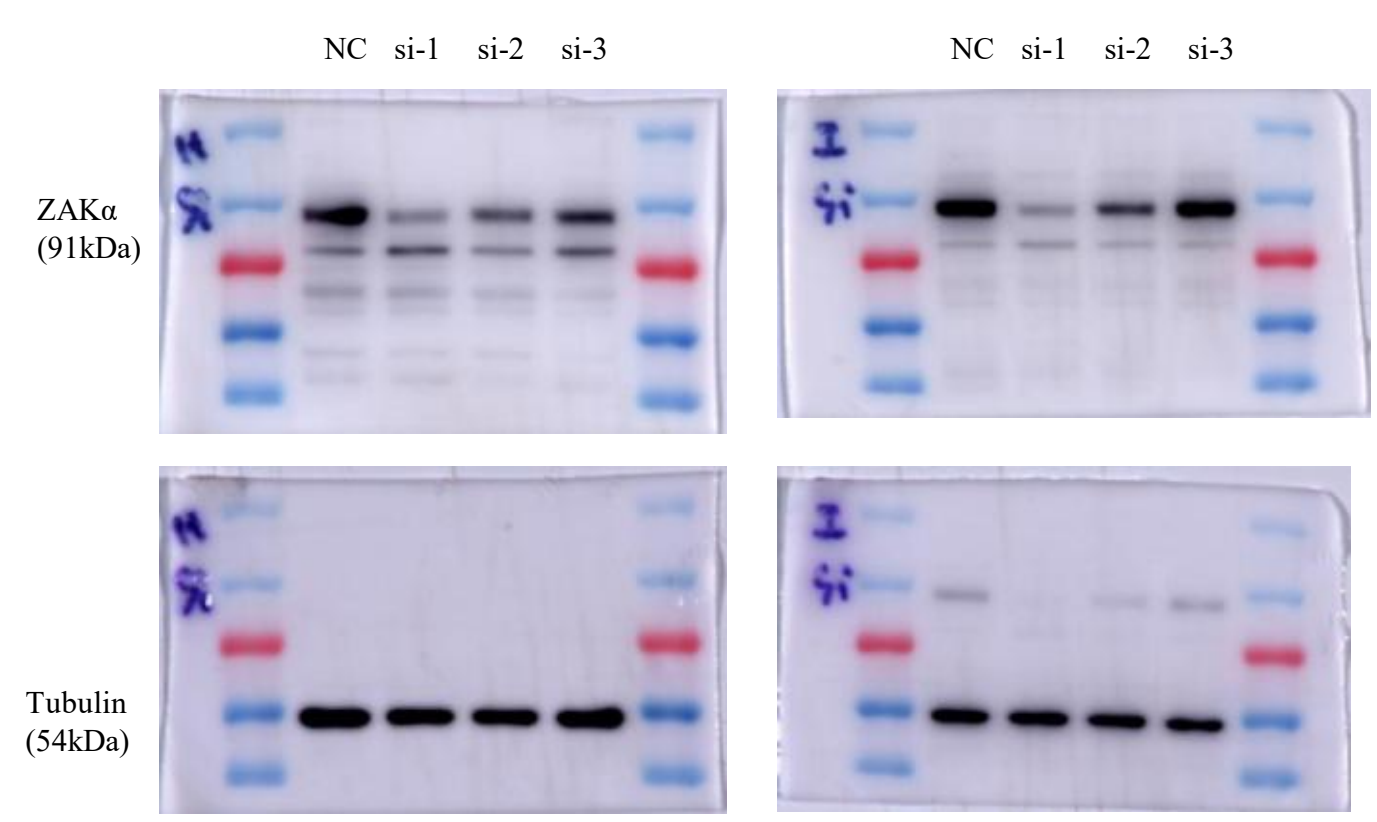

Figure 4J

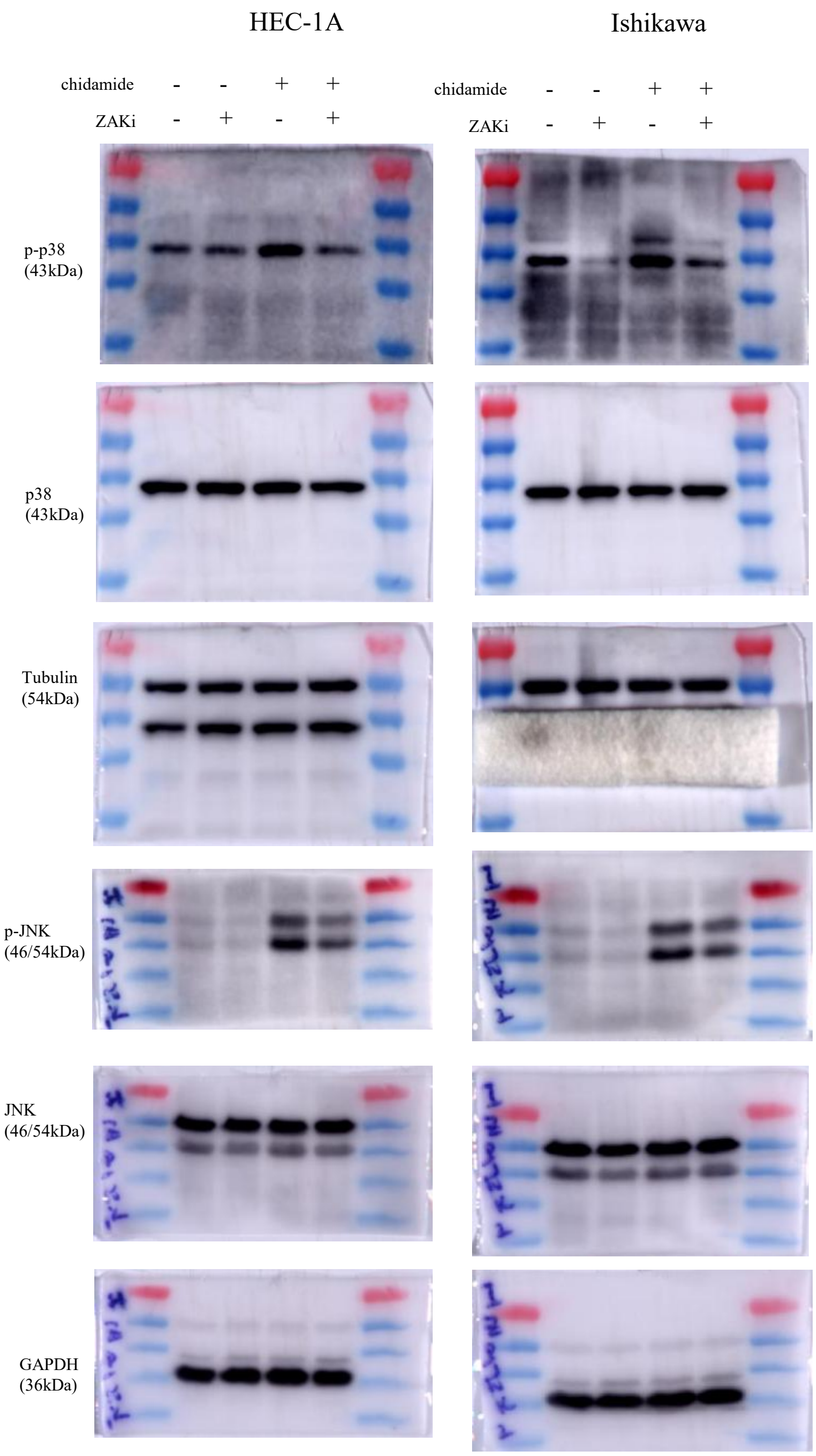

Figure 4K

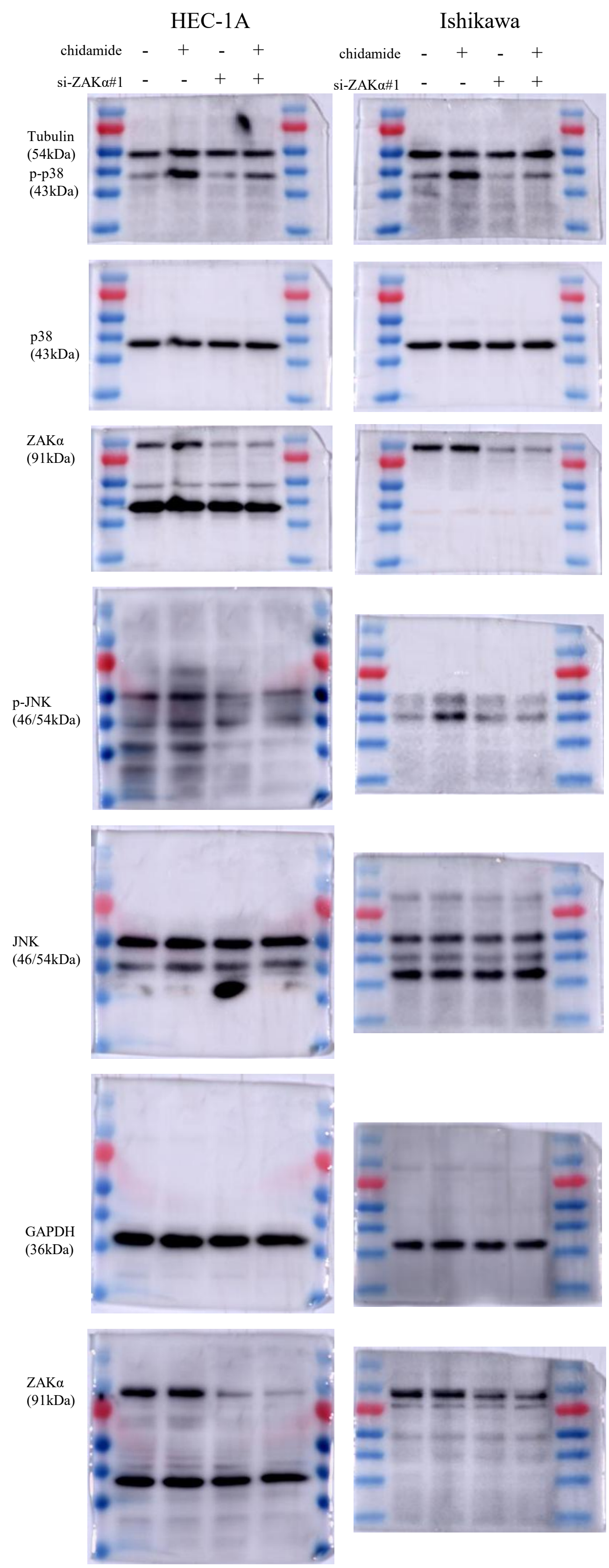

Figure 5A

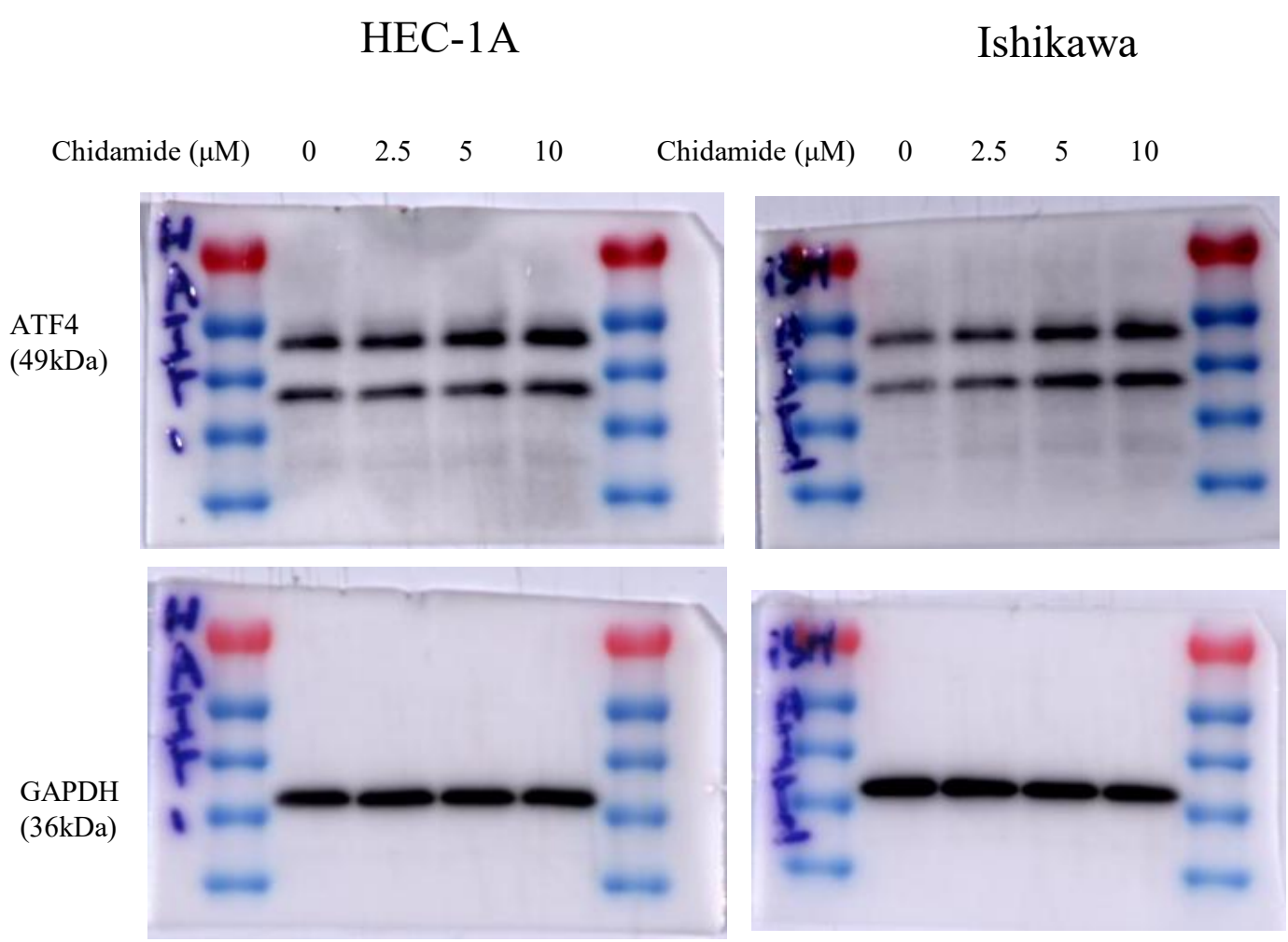

Figure 5C

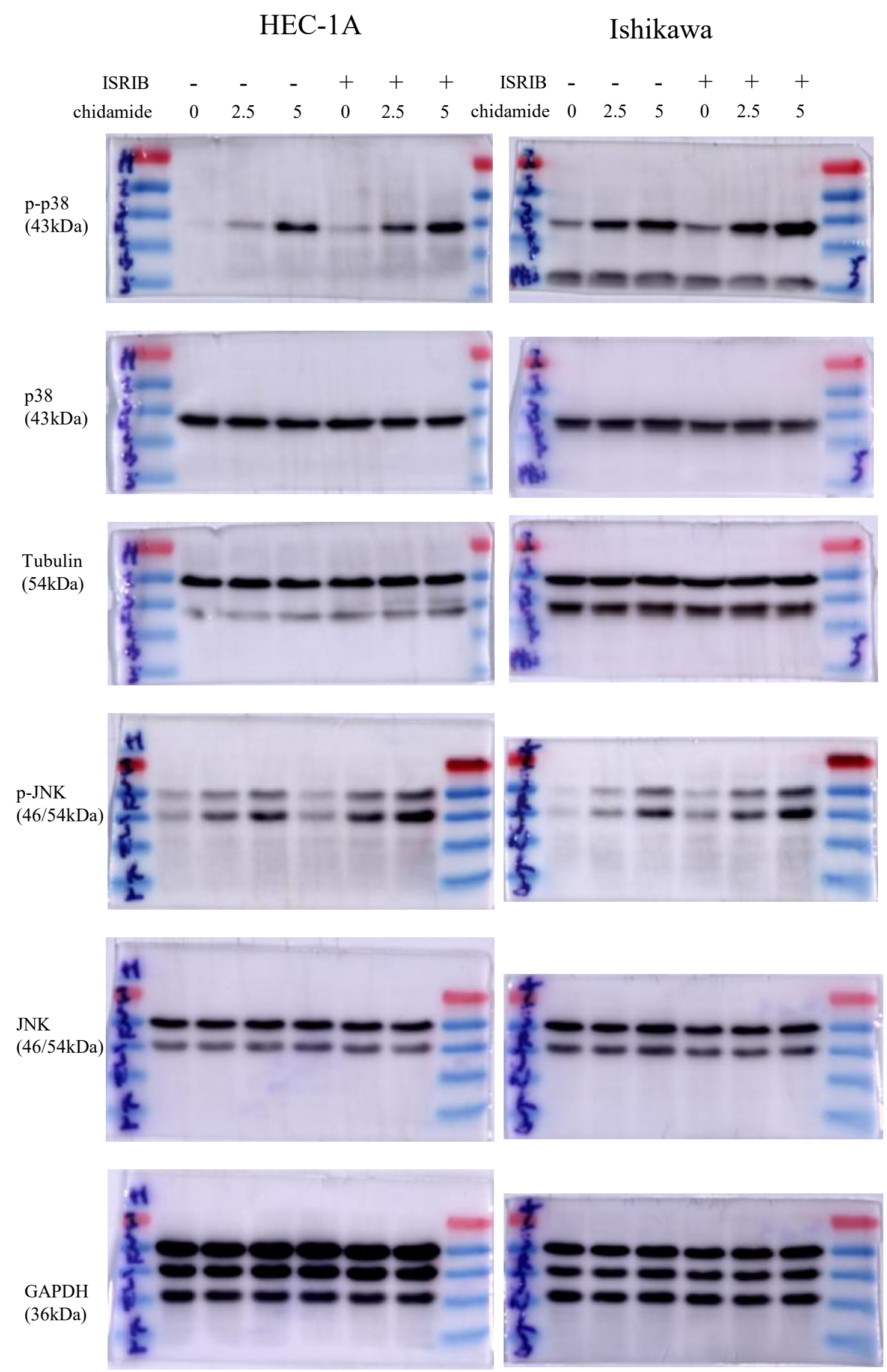

Figure 5B

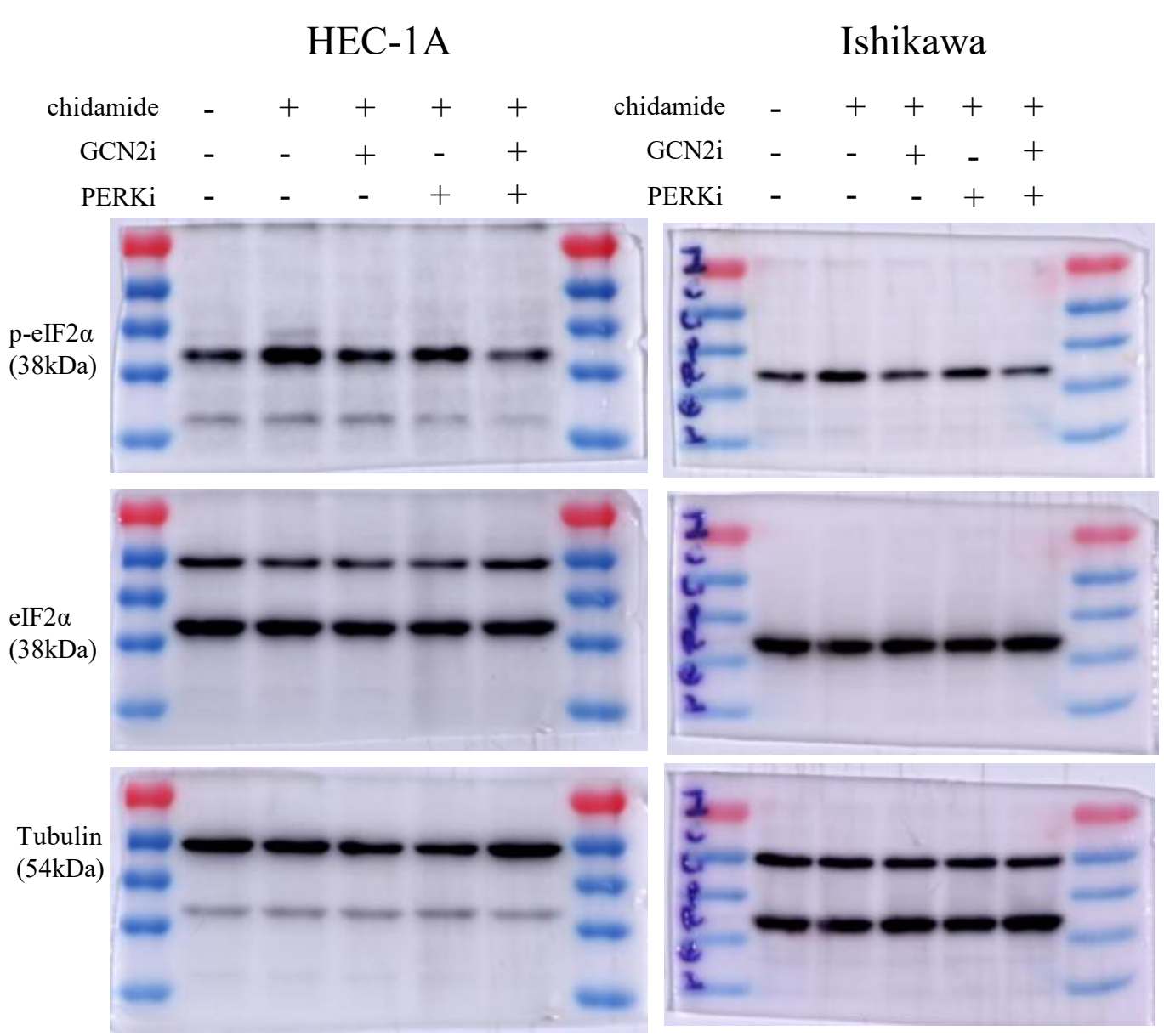

Figure 5D

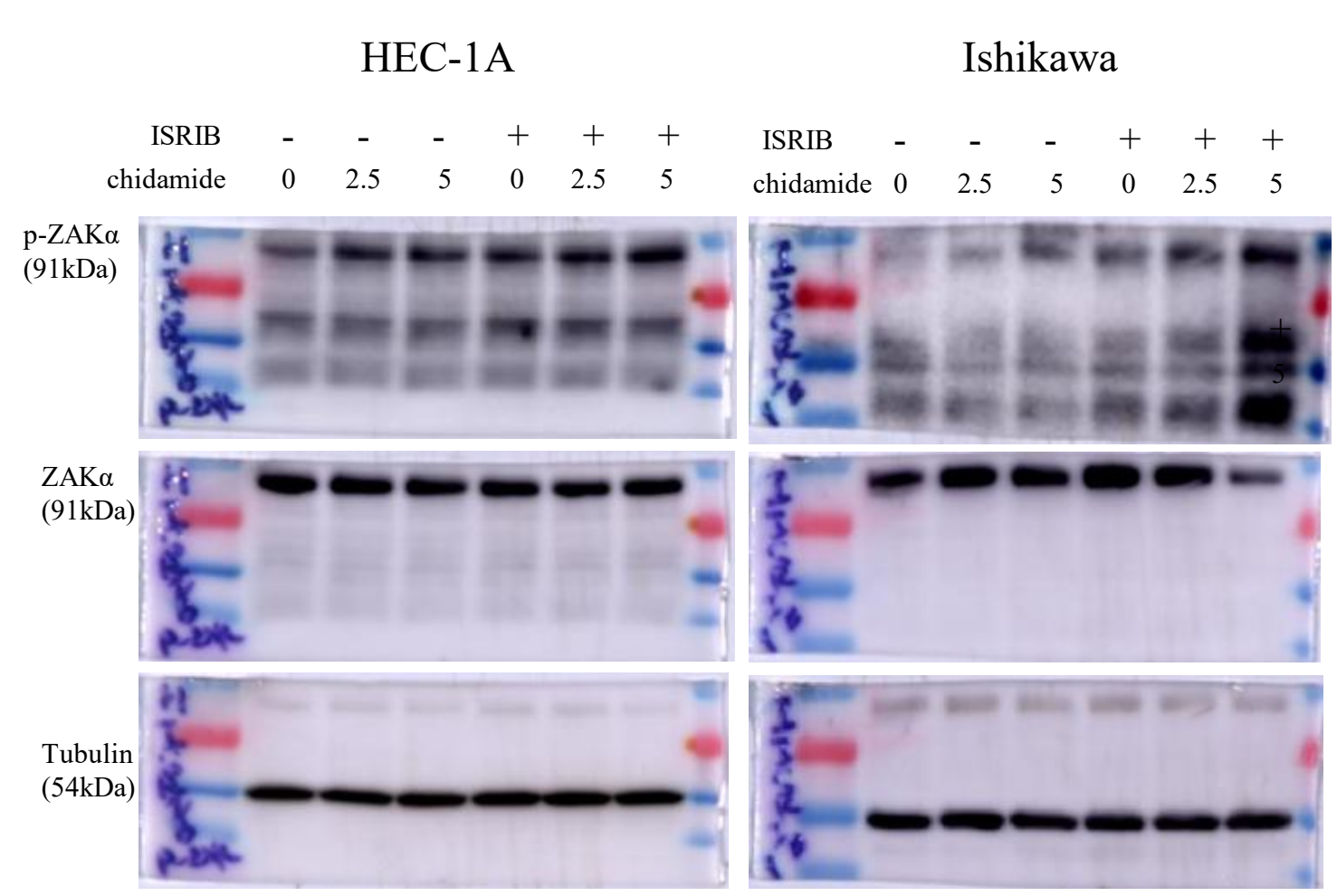

Figure 5E

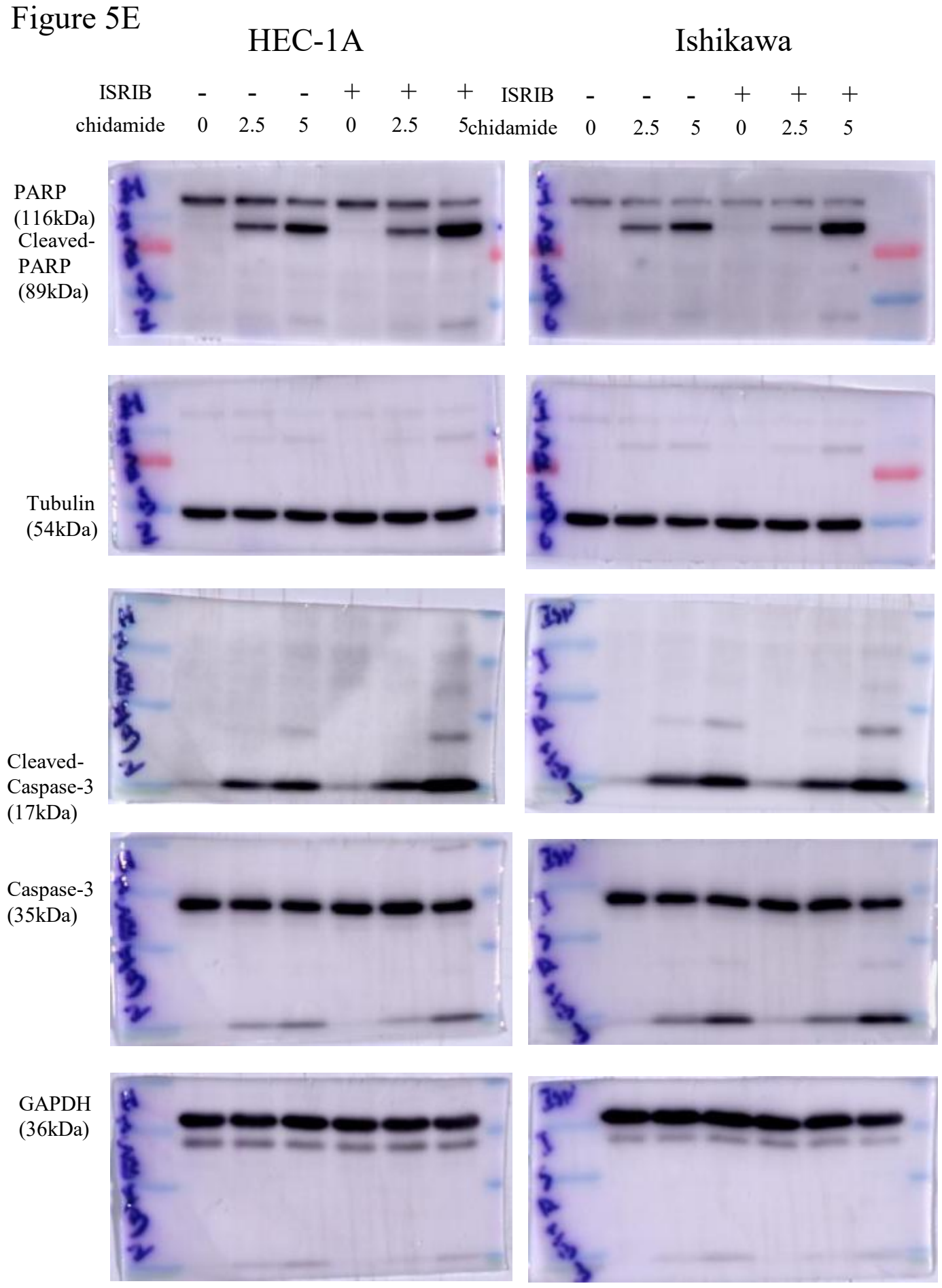

Figure S1, B

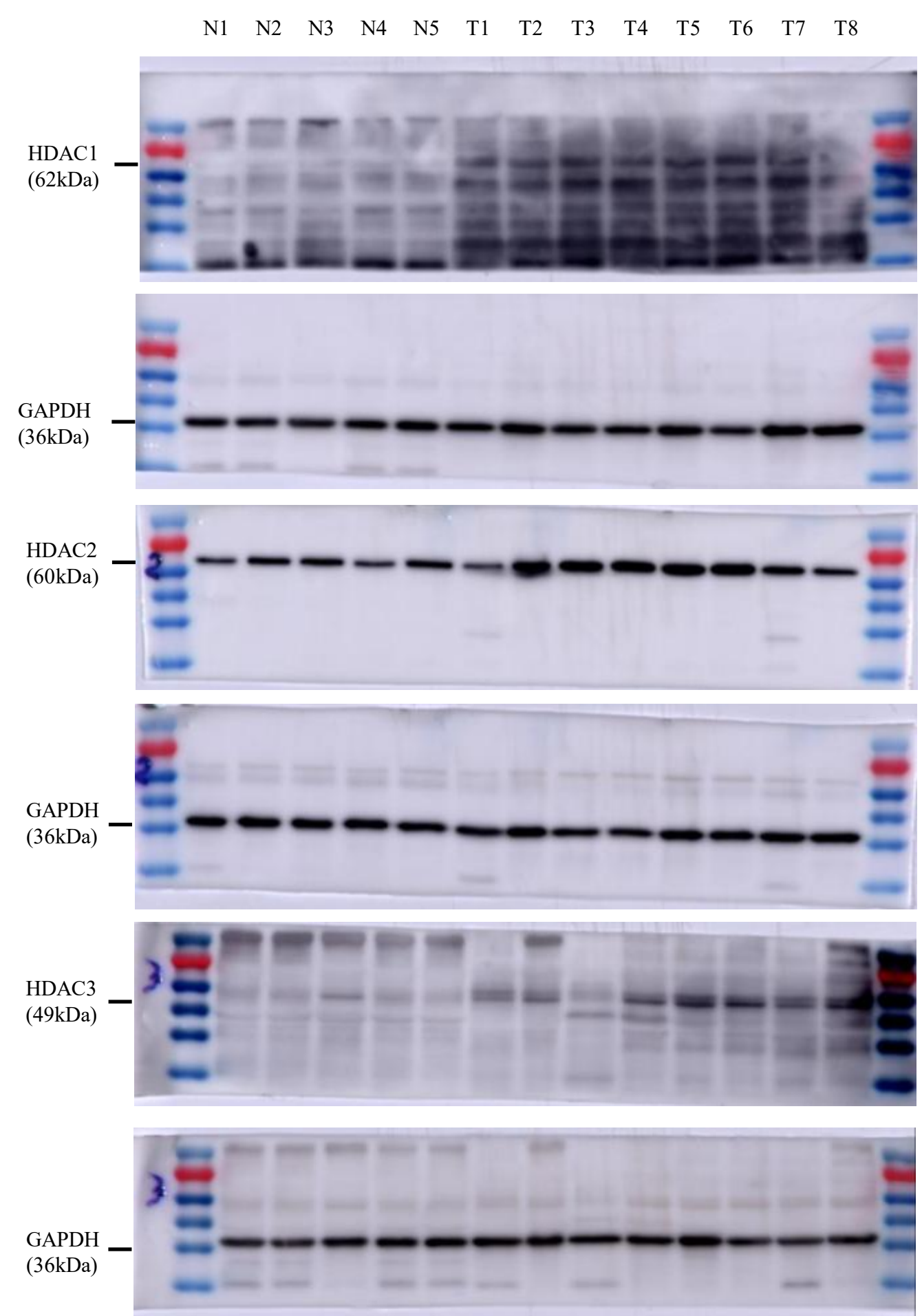

Figure S4G

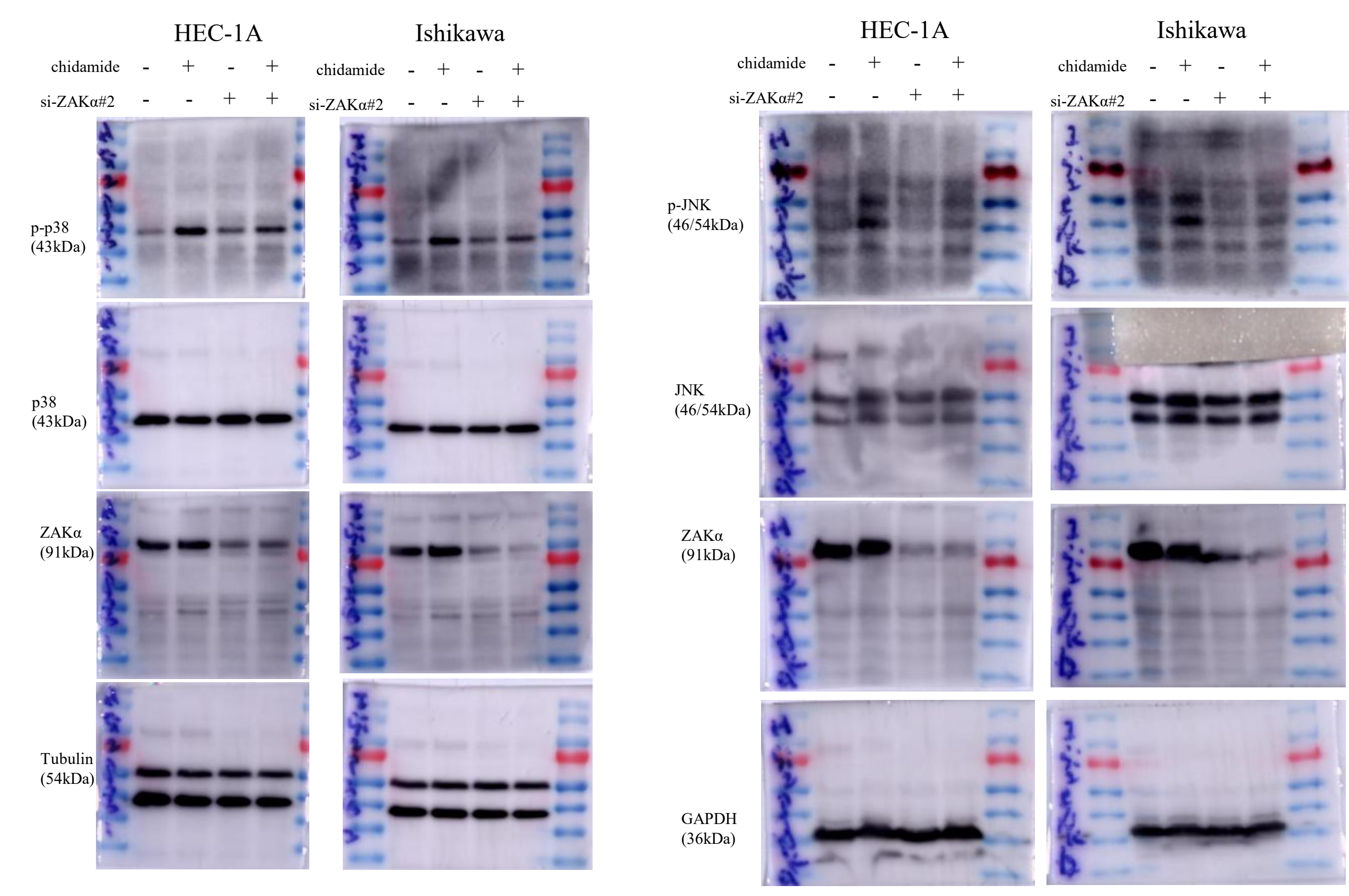

Supplement: Supplementary file 1 [file 41419_2026_9125_MOESM7_ESM.pdf]
