# Supplementary material for: Chidamide induces ROS-driven ZAKα-dependent ribotoxic stress in endometrial cancer and enhances radiosensitivity in vitro and in vivo
Source: Cell Death Dis. 2026 Jul 23;17(1):892. doi: 10.1038/s41419-026-09125-x (PMC13680392; doi:10.1038/s41419-026-09125-x)
Supplement: Supplementary file 2 [file 41419_2026_9125_MOESM1_ESM.docx]

**Supplementary Figure Legends**

Fig. S1 HDAC expression landscape in EC and supplementary validation of the antitumor effects of chidamide. A Comparative expression analysis of HDAC1, HDAC2, and HDAC3 in EC and normal endometrial tissues. B Western blot analysis of HDAC1, HDAC2, and HDAC3 protein levels in fresh-frozen tissues from patients with normal endometria (N, n = 5) and EC (T, n = 8). C Quantification of protein band intensities corresponding to Fig. S1B. D Analysis of the TCGA cohort revealed a correlation between high and low HDAC1 expression and PFS (high HDAC1 group = 264, low HDAC1 group = 280) and OS (high HDAC1 group = 202, low HDAC1 group = 342) in patients with endometrial cancer. E Analysis of the TCGA cohort revealed a correlation between high and low HDAC2 expression and PFS (high HDAC2 group = 143, low HDAC2 group = 401) and OS (high HDAC2 group = 181, low HDAC2 group = 363) in patients with EC. F Analysis of the TCGA cohort revealed a correlation between high and low HDAC3 expression and PFS (high HDAC3 group = 137, low HDAC3 group = 407) and OS (high HDAC3 group = 333, low HDAC3 group = 211) in patients with endometrial cancer. G Colony formation assays evaluating the effects of chidamide on the proliferative capacity of AN3CA, HEC-1A, Ishikawa, and RL95-2 cells with quantification (n = 3 in each group). H Quantification of apoptosis-related protein levels corresponding to the representative Western blots shown in Fig. 1B (n = 3 in each group). I Quantification of cell cycle-related protein levels corresponding to the representative Western blots shown in Fig. 1C (n = 3 in each group). J Quantification of γ-H2AX levels corresponding to the representative Western blots shown in Fig. 1D (n = 3 in each group). K Immunofluorescent labeling of the DNA damage marker protein γ-H2AX to detect DNA damage in cells exposed to chidamide. L Quantification analysis of fluorescent intensity from Fig. S1K (n = 3 in each group). ns: no significance, * *p* < 0.05, ** *p* < 0.01, *** *p* < 0.001, **** *p* < 0.0001.

**Fig. S2** Supplementary RNA-seq analysis and quantitative assessment of chidamide-induced translational impairment and stress signaling. **A** Volcano plot of RNA-seq in Ishikawa cells. **B** Rich factor bubble plot showing top 15 terms in GO enrichment analysis of downregulated DEGs between NC and chidamide-treated groups of Ishikawa cells. **C** Rich factor bubble plot of top 15 pathways in KEGG enrichment analysis of total DEGs between NC and chidamide-treated groups of Ishikawa cells. **D** Quantification of cleaved-PARP/PARP ratios and global protein synthesis, as assessed by puromycin incorporation in the SUnSET assay, corresponding to the representative Western blots shown in Fig. 2F (n = 3 in each group). **E** Quantification of puromycin incorporation in the HTN-based ribosome run-off assay corresponding to the representative Western blots shown in Fig. 2G. Puromycin signals were normalized to the corresponding HTN(-) condition within each treatment group to assess the relative decline in ribosome run-off over time (n = 3 in each group). **F, G** Quantification of p-ZAKα/ZAKα, p-p38/p38, p-JNK/JNK and p-eIF2α/eIF2α levels in chidamide-treated HEC-1A and Ishikawa cells, respectively, corresponding to the representative Western blots shown in Fig. 2H (n = 3 in each group). ns: no significance, * *p* < 0.05, ** *p* < 0.01, *** *p* < 0.001, **** *p* < 0.0001.

**Fig. S3** Quantitative analysis of Western blot results related to NAC-mediated attenuation of chidamide-induced translational stress and downstream signaling. **A** Quantification of puromycin incorporation in the SUnSET assay following NAC pretreatment and subsequent chidamide treatment, corresponding to the representative Western blots shown in Fig. 3C (n = 3 in each group). **B,** **C** Quantification of p-ZAKα/ZAKα, p-p38/p38, p-JNK/JNK and p-eIF2α/eIF2α levels in HEC-1A and Ishikawa cells following NAC pretreatment and subsequent chidamide treatment, corresponding to the representative Western blots shown in Fig. 3D (n = 3 in each group). **D,** **E** Quantification of cleaved-PARP/PARP, Bax/Bcl-2 and cleaved Caspase-3/Caspase-3 ratios in HEC-1A and Ishikawa cells following NAC pretreatment and subsequent chidamide treatment, corresponding to the representative Western blots shown in Fig. 3I (n = 3 in each group). **F,** **G** Quantification of p-CDK1^T161^/CDK1 and Cyclin B1 levels in HEC-1A and Ishikawa cells following NAC pretreatment and subsequent chidamide treatment, corresponding to the representative Western blots shown in Fig. 3J (n = 3 in each group). ns: no significance, * *p* < 0.05, ** *p* < 0.01, *** *p* < 0.001, **** *p* < 0.0001.

**Fig. S4** Quantitative validation of ZAK inhibition and ZAKα knockdown in attenuating chidamide-induced apoptosis, cell cycle alteration and MAPK activation. **A** Quantification of cleaved-PARP/PARP, Bax/Bcl-2 and cleaved Caspase-3/Caspase-3 ratios following ZAKi pretreatment and subsequent chidamide treatment, corresponding to the representative Western blots shown in Fig. 4E (n = 3 in each group). **B** Quantification of p-CDK1^T161^/CDK1 and Cyclin B1 levels following ZAKi pretreatment and subsequent chidamide treatment, corresponding to the representative Western blots shown in Fig. 4F (n = 3 in each group). **C** Quantification of ZAKα knockdown efficiency using three independent siRNAs, corresponding to the representative Western blots shown in Fig. 4G (n = 3 in each group). **D** Representative flow cytometry plots and quantification of apoptosis in chidamide-treated cells after transfection with si-ZAKα#2 (n = 3 in each group). **E** Quantification of p-p38/p38 and p-JNK/JNK levels following ZAKi pretreatment and subsequent chidamide treatment, corresponding to the representative Western blots shown in Fig. 4J (n = 3 in each group). **F** Quantification of p-p38/p38 and p-JNK/JNK levels after transfection with si-ZAKα#1 followed by chidamide treatment, corresponding to the representative Western blots shown in Fig. 4K (n = 3 in each group). **G** Representative Western blots showing changes in p-p38/p38 and p-JNK/JNK levels in HEC-1A and Ishikawa cells after transfection with si-ZAKα#2 followed by chidamide treatment. **H** Quantification of p-p38/p38 and p-JNK/JNK levels corresponding to the representative Western blots shown in Fig. S4G (n = 3 in each group). ns: no significance, * *p* < 0.05, ** *p* < 0.01, *** *p* < 0.001, **** *p* < 0.0001.

**Fig. S5** Quantification of Western blot analyses related to ISR modulation of chidamide-induced stress signaling and apoptosis. **A** Quantification of ATF4 expression in endometrial cancer cells following chidamide treatment, corresponding to the representative Western blots shown in Fig. 5A (n = 3 in each group). **B** Quantification of p-eIF2α/eIF2α levels in cells pretreated with GCN2 inhibitor and/or PERK inhibitor followed by chidamide treatment, corresponding to the representative Western blots shown in Fig. 5B (n = 3 in each group). **C** Quantification of p-p38/p38 and p-JNK/JNK levels in HEC-1A and Ishikawa cells pretreated with DMSO or ISRIB followed by chidamide treatment, corresponding to the representative Western blots shown in Fig. 5C (n = 3 in each group). **D** Quantification of p-ZAKα/ZAKα levels in HEC-1A and Ishikawa cells pretreated with DMSO or ISRIB followed by treatment with the indicated concentrations of chidamide, corresponding to the representative Western blots shown in Fig. 5D (n = 3 in each group). **E** Quantification of apoptosis-associated protein levels in HEC-1A and Ishikawa cells pretreated with DMSO or ISRIB followed by chidamide treatment, corresponding to the representative Western blots shown in Fig. 5E (n = 3 in each group). ns: no significance, * *p* < 0.05, ** *p* < 0.01, *** *p* < 0.001, **** *p* < 0.0001.
