# Supplementary material for: Chidamide induces ROS-driven ZAKα-dependent ribotoxic stress in endometrial cancer and enhances radiosensitivity in vitro and in vivo
Source: Cell Death Dis. 2026 Jul 23;17(1):892. doi: 10.1038/s41419-026-09125-x (PMC13680392; doi:10.1038/s41419-026-09125-x)
Supplement: Supplementary file 3 [file 41419_2026_9125_MOESM8_ESM.docx]

**Supplementary tables**

**Table S1 and S2**

Table S1. Antibodies used in this study

| Name | Company | Cat.number | | RRID | Usage and dilution ratio |
| --- | --- | --- | --- | --- | --- |
| anti-HDAC1 | Santa Cruz | | sc-81598 | AB_2118083 | WB 1:2000 |
| anti-HDAC2 | Santa Cruz | | sc-9959 | AB_627704 | WB 1:2000 |
| anti-HDAC3 | Santa Cruz | | sc-81600 | AB_2118720 | WB 1:2000 |
| anti-phospho-p38 | CST | | 4511 | AB_2139682 | WB 1:1000  IHC 1:600 |
| anti-phospho-JNK | CST | | 4668 | AB_823588 | WB 1:1000  IHC 1:200 |
| anti-p38 | CST | | 9212 | AB_330713 | WB 1:3000 |
| anti-SAPK/JNK | CST | | 9252 | AB_2250373 | WB 1:3000 |
| anti-phospho-ZAK | ImmunoWay | | YP1725 | AB_3718053 | WB 1:1000 |
| anti-ZAKα | Bethyl | | A301-993A | AB_1576612 | WB 1:2000 |
| anti-phospho-eIF2α | CST | | 3398 | AB_2096481 | WB 1:2000 |
| anti-eIF2α | CST | | 9722 | AB_2230924 | WB 1:3000 |
| anti-puromycin | Millipore | | MABE343 | AB_2566826 | WB 1:10000 |
| anti-γ-H_2_AX | Abcam | | ab81299 | AB_1640564 | WB 1:10000  IHC 1:250 |
| anti-Cleaved PARP | CST | | 5625 | AB_10699459 | WB 1:1000 |
| anti-PARP | CST | | 9542 | AB_2160739 | WB 1:1000 |
| anti-Cleaved Caspase-3 | CST | | 9661 | AB_2341188 | WB 1:1000  IHC 1:400 |
| anti-Caspase-3 | CST | | 9662 | AB_331439 | WB 1:1000 |
| anti-Bcl-2 | ABclonal | | A19693 | AB_2862738 | WB 1:1000 |
| anti-Bax | ABclonal | | A19684 | AB_2862733 | WB 1:1000 |
| anti-Cyclin B1 | CST | | 4138 | AB_2072132 | WB 1:1000 |
| anti-Phospho-cdc2^Thr161^ | CST | | 9114 | AB_2074652 | WB 1:1000 |
| anti-Cdc2 | CST | | 77055 | AB_2716331 | WB 1:1000 |
| anti-ATF4 | CST | | 11815 | AB_2616025 | WB 1:1000 |
| anti-α-tubulin | Proteintech | | 80762-1-RR | AB_2918911 | WB 1:50000 |
| anti-GAPDH | Proteintech | | 60004-1-Ig | AB_2107436 | WB 1:100000 |
| anti-rabbit IgG | CST | | 7074 | AB_2099233 | WB 1:5000 |
| anti-mouse IgG | CST | | 7076 | AB_330924 | WB 1:5000 |
| anti-PR | Abcam | | ab32085 | AB_777452 | IF 1:250 |
| anti-ER | Abcam | | ab32063 | AB_732249 | IF 1:500 |
| anti-p53 | Santa Cruz | | sc-126 | AB_628082 | IF 1:100 |
| anti-CK7 | Abcam | | ab68459 | AB_1139824 | IF 1:500 |
| anti-EpCAM | Abcam | | ab213500 | AB_2884975 | IF 1:8000 |
| anti-Ki-67 | Abcam | | ab15580 | AB_443209 | IHC 1:200 |

Table S2. Cells used in this study

| Cell | Source | Cat.number |
| --- | --- | --- |
| AN3CA | Chinese Academy of Sciences | #TCHu246;  RRID: CVCL_0028 |
| HEC-1A | Chinese Academy of Sciences | #TCHu149;  RRID: CVCL_0293 |
| Ishikawa | ECACC | #99040201;  RRID: CVCL_2529 |
| RL95-2 | Chinese Academy of Sciences | #TCHu198;  RRID: CVCL_0505 |

Cell lines were authenticated by STR analyses. Mycoplasma contamination was routinely tested and tests were negative.
